# *CDH1* Gene Mutation Hereditary Diffuse Gastric Cancer Outcomes: Analysis of a Large Cohort, Systematic Review of Endoscopic Surveillance, and Secondary Cancer Risk Postulation

**DOI:** 10.3390/cancers13112622

**Published:** 2021-05-26

**Authors:** Matthew G. K. Benesch, Stuart R. Bursey, Andrew C. O’Connell, Morag G. Ryan, Carrie L. Howard, Cecily C. Stockley, Alexander Mathieson

**Affiliations:** 1 Discipline of Surgery, Faculty of Medicine, Memorial University of Newfoundland, St. John’s, NL A1B 3V6, Canada; aoconnell@mun.ca (A.C.O.); carrieh@mun.ca (C.L.H.); ccs106@mun.ca (C.C.S.); alex.mathieson@med.mun.ca (A.M.); 2 Faculty of Medicine, Memorial University of Newfoundland, St. John’s, NL A1B 3V6, Canada; srb312@mun.ca; 3 Discipline of Family Medicine, Faculty of Medicine, Memorial University of Newfoundland, St. John’s, NL A1B 3V6, Canada; morag.g.ryan@mun.ca

**Keywords:** *CDH1*, E-cadherin, mutation, gastric cancer, lobular breast cancer, Cambridge Protocol, cancer risk

## Abstract

**Simple Summary:**

Some patients carry a mutated copy of the *CDH1* gene that can lead to a very rare form of hereditary gastric cancer called signet-ring cell adenocarcinoma (SRCC). SRCCs rarely form visible tumors prior to spreading. Hence, patients are recommended to have prophylactic gastrectomies at a young age. Many patients wish to avoid surgery and thus have regular checks with upper endoscopy with biopsies to rule out cancer. Further, these patients may also be at risk of other cancers beyond the already known breast cancer risks, but this is not known. In this study, we show that despite systematic biopsy protocols, many early cancers might be missed on endoscopy. Therefore, patients should not rely on endoscopy to delay surgery. These patients may also be at increased risk of colorectal SRCC, which has very poor survival outcomes. To confirm this, we need a central database that captures outcomes for this patient population.

**Abstract:**

Hereditary diffuse gastric cancer (HDGC) is a rare signet-ring cell adenocarcinoma (SRCC) linked to *CDH1* (E-cadherin) inactivating germline mutations, and increasingly other gene mutations. Female *CDH1* mutation carriers have additional risk of lobular breast cancer. Risk management includes prophylactic total gastrectomy (PTG). The utility of endoscopic surveillance is unclear, as early disease lacks macroscopic lesions. The current systematic biopsy protocols have unknown efficacy, and other secondary cancer risks are postulated. We conducted a retrospective study of consecutive asymptomatic HDGC patients undergoing PTG, detailing endoscopic, pathologic, and outcome results. A systematic review compared endoscopic biopsy foci detection via random sampling versus Cambridge Protocol against PTG findings. A population-level secondary-cancer-risk postulation among sporadic gastric SRCC patients was completed using the Surveillance, Epidemiology, and End Results database. Of 97 patients, 67 underwent PTG, with 25% having foci detection on random endoscopic biopsy despite 75% having foci on final pathology. There was no improvement in the endoscopic detection rate by Cambridge Protocol. The postulated hazard ratio among sporadic gastric SRCC patients for a secondary colorectal SRCC was three-fold higher, relative to conventional adenocarcinoma patients. Overall, HDGC patients should not rely on endoscopic surveillance to delay PTG, and may have secondary SRCC risks. A definitive determination of actual risk requires collaborative patient outcome data banking.

## 1. Introduction

Hereditary diffuse gastric cancer (HDGC) accounts for 1–3% of all gastric cancer diagnoses [1]. It is an autosomal dominant condition with incomplete penetrance primarily associated with inactivating mutations in *CDH1* (E-cadherin). This germline gene mutation was first described in 1994 and was confirmed to be responsible for HDGC in 1998 [2]. These patients have a 70% lifetime risk of gastric cancer in males and 56% in females with a median diagnosis age of 38 years (range 14–69 years) [3,4]. Women additionally have a 42% lifetime risk of lobular breast cancer [3]. The current management for identified carriers includes prophylactic total gastrectomy (PTG) between the ages of 20–40 years, and the initiation of high-risk breast cancer screening with annual mammography and MRI at age 30–35 years for female carriers [5].

The genetic counseling and testing criteria for *CDH1* mutations were updated in 2019, with full criteria indications for patients with two or more documented cases of gastric cancer at any age in first- or second-degree relatives with at least once confirmed DGC, personal history of DGC before age 40 years, or personal or family history (first- or second-degree relatives) of DGC and lobular breast cancer with at least one diagnosed before the age of 50 years [6,7]. Additionally, testing for the *CDH1* mutation is supported in families with bilateral or multiple cases of lobular breast cancer before the age of 50 years, families with a clustering of DGC and cleft lip/cleft palate, or any patient diagnosed with gastric in situ signet-ring cells and/or pagetoid spread of signet-ring cells [6,7]. Associations between HDGC and other genes are now being discovered, which may explain why 11% of HDGC cases arise in patients who are negative for *CDH1* mutations [3]. These patients instead may have mutations in possibly associated genes *CTNNA1, BRCA2, STK11, SDHB, PRSS1, ATM, MSR1*, and *PALB2* [3]. In even more recently revised clinical practice guidelines by Blair et al., HDGC is now defined by the presence of a pathogenic germline *CDH1* or *CTNNA1* variant [8]. *CTNNA1* encodes for another adherens junction protein, αE-catenin, that is also found in a small minority of HDGC cases [9]. For patients that meet the criteria indications for genetic testing, consideration for *CTNNA1* analysis is warranted if no *CDH1* pathological variant is found [8]. Further work on the penetrance of *CTNNA1* is required, as well as its implication in lobular breast cancer risk [8]. 

Physiologically, E-cadherin as a transmembrane glycoprotein has numerous signaling pathway roles, including mediating cell adhesions and polarity [10]. As a tumor suppressor, it is downregulated among the initiating steps of the epithelial-mesenchymal transition resulting in cellular plasticity and a migratory phenotype required for metastatic disease [10]. Many described mutations result in protein truncation leading to protein nonfunction, not all of which have an appreciated clinical significance [3,11,12]. Upwards of 20% of now known pathologic *CDH1* mutations are missense [11,12,13]. The functional relevance of these mutations is an active area of investigation, because in these mutations, normal protein length and expression levels are typically observed [11]. Because *CDH1* is a tumor suppressor gene, usually a second somatic hit is required for tumor initiation, which typically involves promoter methylation [14]. 

*CDH1* gastric cancers present as signet-ring cell adenocarcinomas (SRCCs) with abundant intracellular mucin, and readily metastasize before forming significant macroscopic primary lesions, accounting for their typical late stage at detection [15]. It is not clear either why *CDH1* mutations are linked primarily with gastric cancer and an increased risk of lobular breast cancer, although case reports document the co-existence of *CDH1* gastric SRCC with colorectal, appendiceal, and pancreatic cancers [16,17,18]. Overall, the evidence is lacking to support routine enhanced cancer screening, especially colorectal, in *CDH1* mutation families [8,19], unless there is a family history of first- and second-degree relatives with a colorectal histopathology showing a mucinous component and/or signet-ring cells [6].

The decision to proceed to PTG should be careful and deliberate. The optimized timing for a PTG from cancer risk and quality-of-life perspectives has been studied over the past decade. Using quality-adjusted life-years (QALYs) as a primary outcome, the optimal age of resection is 39 years in men and 30 years in women [20]. Health-related quality-of-life parameters often decrease immediately after surgery, which slowly recover and often remain below preoperative baseline levels [21]. Patients have on average 19% weight loss after surgery that does not recover, and they require lifelong management of micronutrient deficiencies [22]. In addition, there are now over 100 clinically relevant *CDH1* mutations with varying penetrance rates [23], all of which are factors that contribute to personal decision-making surrounding the timing of surgery. Unfortunately, given the microscopic nature of diffuse cancer foci, there are no good surveillance tests, but patients wishing to delay surgery are recommended to undergo regular endoscopic surveillance with biopsies [6,8]. Unfortunately, a comprehensive review of 174 patients undergoing random biopsies had a detection rate of only 28.3%, despite cancer foci being found in 87.4% of these patients after PTG [24]. Additional adjuncts including chromoendoscopy and endoscopic ultrasound have been investigated to improve detection rates with no additional utility [25,26]. The current consensus recommendation for endoscopic surveillance is the Cambridge Protocol that employs a systematic examination of the stomach with 30 biopsies with five each from the prepyloric area, antrum, transition zone, body, fundus, and cardia, in addition to any targeted biopsies of suspicious gastric abnormalities [1,6]. It is not known if this protocol improves foci detection rates in asymptomatic patients over random biopsies, as there have been no direct comparisons between the two protocols. 

The province of Newfoundland and Labrador in eastern Canada has one of the largest known cohorts of *CDH1* germline mutation carriers in the world. This cohort was first described in 2009 after the first 23 PTGs were completed [27]. In this paper, we present findings from three aims of investigation. First, we provide an update to the endoscopic, surgical, and surveillance outcomes of an expanded cohort of 97 eligible patients in the Newfoundland and Labrador cohort. Second, we perform a systematic review comparing the detection rate of cancer foci via endoscopy with random biopsies against the standardized Cambridge Protocol in asymptomatic patients with a documented *CDH1* mutation. Third, in lieu of any comprehensive database that tracks outcomes of *CDH1* mutation carriers, we calculate an estimated secondary cancer risk among patients with sporadic gastric SRCCs, using the Surveillance, Epidemiology, and End Results (SEER) database. This aim is conducted to postulate whether our findings of unusual secondary cancers in *CDH1* mutation patients in Newfoundland and Labrador might warrant careful consideration of other secondary cancer risks in other *CDH1* mutation cohorts. 

## 2. Materials and Methods

### 2.1. Newfoundland and Labrador Cohort Design

We identified all patients with a genetically confirmed *CDH1* mutation in Newfoundland and Labrador from 2002 to January 2019 via our local Provincial Medical Genetics Program and included all patients who were either clinically asymptomatic at the time of their PTG or otherwise under active surveillance through to August 2020. Board-certified gastroenterologists or general surgeons performed all endoscopies. General surgeons in Newfoundland and Labrador, Ontario, or Alberta (Canada) performed all PTGs. All biopsies and pathological specimens were prepared and examined by Canadian board-certified pathologists with expertise in gastrointestinal malignancies. Medical charts were reviewed using a standardized data extraction form. 

Ethics approval for this aim was obtained from the Memorial University of Newfoundland Health Research Ethics Board (HREB #2018.215) prior to the commencement of this study. Because data was collected from patient charts for secondary use under an ethics board approval, explicit individual patient consent was not required. 

### 2.2. Systematic Review of Random Endoscopic Biopsies versus the Cambridge Protocol Approach 

MEDLINE and Embase were searched from inception to 31 August 2020 (Table S1) without language restriction using a prospectively registered PROSPERO protocol (CRD42020184631). We identified studies (including conference abstracts) correlating endoscopic biopsy results (cancer positive or negative) obtained randomly (no systematic methodology employed or described) or via the Cambridge Protocol to final pathology results (cancer positive or negative) in asymptomatic patients with a known *CDH1* mutation. 

Two reviewers independently assessed all citations for eligibility, based on our protocol criteria (Table S2) and disagreements were resolved by discussion. If patients were presented in multiple publications, the most recent publication was included in the analysis. Case series were assessed using the Institute of Health Economics Quality Appraisal Checklist for Case Series Studies [28], and case reports using the JBI Critical Appraisal Checklist for Case Reports [29] (Table S3). Studies were considered to have a low risk of bias if at least 80% of criteria were met, moderate risk if at least 60% of criteria were met, and high risk if less than 60% of criteria were met. Two reviewers, who both independently assessed studies for methodological quality, used standardized data extraction forms to capture relevant data (Table S4). To determine a literature estimate of the endoscopic detection rate from random biopsies and Cambridge Protocol, we then correlated the biopsy and final pathology results (cancer positive/negative) from all studies that detailed individual patient information rather than aggregate summaries.

### 2.3. Estimated Secondary Cancer Risk for Patients with a Sporadic Gastric SRCC via the SEER Database

All data from the 18 SEER cancer registries (1975–2016) were used as previously described [15]. Data released from the SEER database do not require informed patient consent. Permission to obtain the SEER database was obtained with the ID number 10095-Nov2018 via signed agreements [30]. Variable definition and data management are described in Tables S5 and S6. Patients with secondary cancers were identified on the basis of the same identification number. A competing-risks regression model of hazard risk ratio of patients with primary SRCC compared to either any other primary cancer or primary adenocarcinoma of the same site was used to compensate for mortality, according to the method of Fine and Gray [31]. Risks were corrected for age and gender. 

### 2.4. Statistical Analysis 

All statistical calculations were performed using Stata 15.1 (StataCorp LLC, College Station, TX, USA). For patient characteristics a Mann–Whitney U-test was used to compare two independent variables with a non-normal distribution, and a χ^2^ test and Fisher’s exact test for nominal variables, as appropriate. All hazard risk ratios were calculated with 95% confidence intervals. All *p*-values were two-sided, and the threshold of 0.05 was used to determine statistical significance.

## 3. Results

We present our results according to the three aims outlined at the end of the Introduction.

### 3.1. Endoscopic, Pathologic, and Outcome Results in the Newfoundland and Labrador Cohort

#### 3.1.1. Characterization of PTG versus No-PTG Patients

All patients in our cohort were seen and counseled by Medical Genetics at Memorial University of Newfoundland and underwent *CDH1* mutation testing, based on the current genetic testing criteria at the time of presentation from 2002 to 2017. In total, we identified 97 consecutive asymptomatic patients diagnosed with a *CDH1* gene mutation in Newfoundland and Labrador. From 2002 to August 2020, 67 patients have undergone PTG (Table 1). There was no statistically significant gender difference between the two groups, but those not having had a PTG had a median age at genetic testing of 51.1 years versus 42.6 years. This no-PTG group also had significantly less follow-up time from genetic testing (4.7 years versus 11.8 years). While the rate of uptake of endoscopic surveillance was equivalent in both groups (80–90%), median endoscopic surveillance time was 1.2 years for PTG patients compared to 4.7 years for no-PTG patients. Of the 67 gastrectomies, 9 had no cancer foci found, 1 had in situ disease, 56 had a T1a cancer, and 1 had a T2a cancer. All perigastric lymph nodes removed via D1 lymphadenectomy were negative. No women in the no-PTG group underwent prophylactic mastectomy, while 44% of women have done so in the PTG group. Overall, three PTG patients have died each from breast, colon SRCC, and pancreatic adenocarcinoma, and two no-PTG patients from rectal cancer and melanoma (Table 1). A total of 17 secondary cancers were observed in our cohort, 9 of which were lobular breast cancers (Table S7).

#### 3.1.2. Characterization of Prophylactic Mastectomy Patients

A total of 17 out of 53 female patients in our Newfoundland and Labrador cohort underwent prophylactic mastectomy for which 16 records were available for review (Table 2). The remaining 36 female patients have either chosen to continue with high-risk breast cancer screening or have discontinued screening due to age or other comorbidities. Nine of these patients elected for bilateral mastectomies with a median age of 41.3 years, compared to 55.2 years for completion mastectomies, at a median time of 10 months following initial breast cancer diagnosis. While not statistically different, patients underwent bilateral mastectomies about 5.9 years after *CDH1* gene mutation diagnosis, but only 2.5 years for completion mastectomies. The final pathology was varied, but lobular breast cancer was found in one patient, and lobular carcinoma in situ (LCIS) in four specimens.

#### 3.1.3. Characterization of Endoscopic Biopsy Results Compared to Final Pathology

Of the 67 patients having undergone PTG, 59 had at least one documented endoscopic examination with biopsy (Table 3). In the biopsy positive group, 93% had cancer on final pathology, and 82% in the biopsy negative group, but this result was not statistically different. A difference in biopsy result did not correlate with gender or mutation type, but patients with a positive biopsy had a median age of 32.3 years compared to 46.8 years for negative biopsies. It took a median of three endoscopes to acquire a positive biopsy diagnosis. The number of biopsies was significantly higher for a positive diagnosis at a median of 18 compared to 13 for the negative diagnosis group. The calculated sensitivity of endoscopic biopsies was 28.0% with a negative predictive value of 18.2%. The total number of foci discovered on final pathology and the method by which specimens were processed (total embedding protocol or representative sampling) were equivalent between the two groups. 

### 3.2. Systematic Review Comparing Random to Cambridge Protocol Endoscopic Biopsies

**Figure 1:**
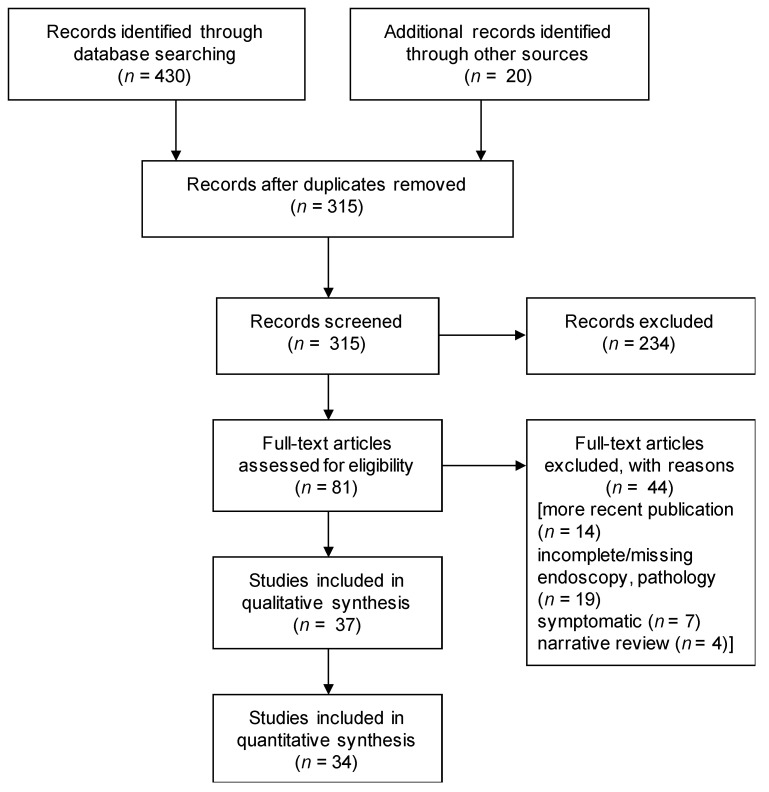
PRISMA study selection flow chart.

Figure 1 presents a PRISMA flow diagram of our literature search summary. We identified 430 records in MEDLINE and Embase and another 20 through checking citations in retrieved results. After the removal of duplicates, 315 records were screened by title and abstracts, with 81 records for a full text review. Forty-four articles were then excluded as indicated (Figure 1). Thirty-seven case reports and case series were included after meeting all inclusion criteria (Table S2) for subsequent bias assessment [16,25,26,32,33,34,35,36,37,38,39,40,41,42,43,44,45,46,47,48,49,50,51,52,53,54,55,56,57,58,59,60,61,62,63,64,65] (Table S3). Our systematic review results are presented in Table S4, with 34 articles eligible for calculating a literature estimate of the endoscopic detection rate of random biopsies and Cambridge Protocol using individual patient data.

From our results, we saw no significant difference in the detection rate of cancer foci by endoscopic biopsy, when compared to the final pathology result after PTG (Table 4). The estimated test sensitivity and negative predictive value for random biopsies were 20.9% and 15.2% respectively, while for Cambridge Protocol, these values were 27.1% and 22.1%, respectively. The median number of biopsies taken in the random group was 14.5, though the range varied from 1–60, and data was only present from 43% of all patients, while all Cambridge Protocol patients had a minimum of 30 biopsies per scope. There were no differences in gender distribution, age at surgery, cancer stage at pathology, total foci count, or embedding protocol technique between the two groups.

### 3.3. Estimation of Secondary Cancer Risk for Patients with a Sporadic Gastric SRCC via the SEER Database

Unfortunately, there is no central database tracking the outcomes of *CDH1*-mutation patients, and therefore counseling patients on secondary cancer risks beyond lobular breast cancer is difficult. Given that all *CDH1*-mutation driven HDGC are SRCCs, and the presence of gastric SRCC is a criterion for *CDH1* testing, we employed the SEER database to estimate a postulated risk of secondary cancers among those with sporadic gastric SRCC relative to patients with either any other type of gastric cancer or conventional gastric adenocarcinomas (Table 5). In total, 172,375 patients, of which 24,226 have SRCC, and 109,397 conventional adenocarcinomas, were eligible for analysis. After competing risk adjustments for death from gastric cancer and adjustments for age and gender, patients with an SRCC were not at overall increased risk for any subsequent cancer diagnosis. We did though confirm an increased risk in lobular breast cancer (1.7-fold), but not ductal, consistent with the literature for *CDH1* patients. These patients were at a 3-fold increased risk of a secondary SRCC cancer, with most cases arising in the colon or rectum.

This analysis was repeated with patients having a primary colorectal cancer diagnosis to examine if this relationship between sporadic gastric and colorectal SRCC was reciprocal (Table 6). Again patients with a primary sporadic colorectal SRCC were still at increased risk of lobular breast cancer (~1.4-fold), and 5-fold increased risk of a secondary gastric SRCC. Finally, we conducted a similar analysis for patients with a sporadic primary lobular breast cancer compared to all other breast cancers and ductal breast cancers (Table 7). Patients with a primary lobular breast cancer were at about 2-fold increased risk of sporadic gastric SRCC and ~1.4-fold of sporadic colorectal SRCC compared to patients with ductal breast cancer.

## 4. Discussion

In this paper we examined the endoscopic, surgical, and surveillance outcomes over nearly 20 years in one of the largest asymptomatic *CDH1* mutation carrier cohorts and highlighted patient management considerations that need further investigation. In particular, we observed that despite the standard of care recommendation for PTG, nearly one-third of our patients have declined PTG. The primary reasons were usually older age at mutation carrier status discovery, which these patients have been genetically fortunate to escape symptomatic disease, and hesitancy regarding proceeding forward with PTG. In our practice, these patients, in accordance with consensus guidelines, underwent at least annual endoscopic surveillance and biopsies [66]. For many of them, a negative biopsy result tended to defer their decision on PTG, whereas a positive biopsy was usually the triggering event for consenting to the procedure. However, it is well known that biopsy effectiveness is poor [24,67], and the lag time between cancer foci development and the risk of symptomatic and potentially incurable disease is not known. Also, in addition to the known lobular breast cancer risk, we observed other secondary cancers including a colorectal SRCC in a 46-year-old patient. Thus, our experiences generated the additional aims of this research, whereby we compared endoscopic effectiveness between historically random biopsies and the now consensus Cambridge Protocol, and estimated potential secondary cancer risks in these patients.

PTG for *CDH1* mutation carriers is a potentially lifesaving intervention, however this surgery and its sequelae are not without morbidity or effects on quality of life [23,68]. Postoperative complications, including anastomotic leaks and strictures, have been reported in up to 46% of patients [22,69]. Quality of life has been routinely correlated to postsurgical outcomes, with up to half of patients expressing decisional regret in the first four weeks following surgery, with slow resolution [21,70]. The advent of multigene panel testing has also led to the discovery of a myriad of *CDH1* mutations, for many of which clinical significance has not been established [71,72,73,74]. Patient counseling requires a multidisciplinary team approach to manage the host of genetic, surveillance, surgical, nutritional, and psychosomatic considerations of this unique population.

Despite *CDH1*-mutation driven HDGC being autosomal dominant in nature, the myriad of mutation types poses two problematic patient counseling concerns. The first is that not all mutations appear to have the same disease penetrance, as some studies report up to 20–30% of patients will not develop gastric cancer [62]. However, these numbers arise from very small cohorts. In a recent large cohort of 95 patients with *CDH1* mutations and a family history of HDGC having undergone PTG, cancer foci were found in 89% of specimens, consistent with our detection rate of 85% [75]. Second, there are large temporal differences in the timing of disease onset, for which some patients would seek a surveillance option to delay surgery for as long as possible. In our cohort of asymptomatic patients, patients choosing to not undergo PTG tended to be older at the time of genetic testing, often being identified on family history following the discovery of the mutation in a younger proband. However, these patients represent a self-selected group that have fortunately not had earlier disease onset. This is also reflected in that those patients with negative pathology on their PTG tended to be older than patients with a positive result (44 versus 34 years old).

Patients choosing not to undergo PTG are recommended to undergo a surveillance endoscopy every 6–12 months. The intention is to detect disease at early stages to maximize the chances of curative surgery. This option is however suboptimal as cancer foci are microscopic with a low endoscopic detection rate, and given the diffuse nature of this cancer, macroscopic tumor formation is rarely appreciated before regional and metastatic spread has occurred. It is estimated that reliable detection of a single cancer foci may require at least 300 biopsies [37]. The natural time course of progression from mucosal cancer foci to fulminant disease is also not known, and given the extremely rare nature of this disease, it is unlikely these kinds of questions can ever be answered. This uncertainty is why patients are recommended to undergo PTG early in adulthood upon genetic confirmation of the mutation [6,7,8].

Consistent with the literature, our large cohort undergoing random surveillance biopsies had a test sensitivity of under 20%. Endoscopic adjuvants to facilitate random biopsy selection have not improved diagnostic yield [25,26]. Attempts to improve this yield through systematic sampling via the Cambridge Protocol have become a new standard by consensus over the last five years. However, in this first systematic review of its kind, we have shown that diagnostic yield is not significantly improved over random biopsies. Therefore, patients choosing surveillance over PTG must be fully informed of the poor and unreliable nature of this test. Systematic biopsy protocols are unlikely to adequately compensate for the inherent inferiority of this surveillance procedure.

**Figure 2:**
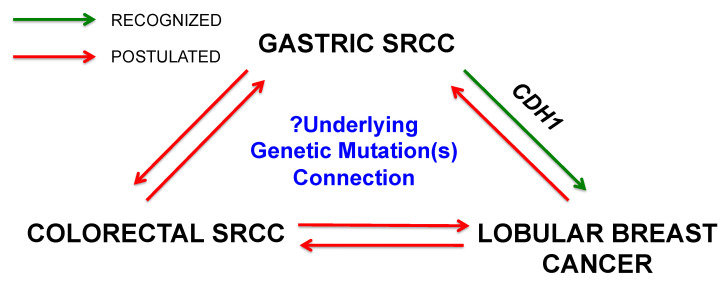
Postulated interrelationship between gastric, colorectal, and lobular breast cancers. The link between gastric SRCC and lobular breast cancer is recognized in *CDH1* germline mutation patients. Our speculative results from sporadic gastric, colorectal, and lobular breast cancer patients (Table 5, Table 6 and Table 7) postulate the existence of bidirectional relationships across all three cancer subtypes that warrant further investigations.

The secondary cancer risk beyond lobular breast cancer has long been suspected [76], but establishing its incidence is limited by the rarity of *CDH1* mutation prevalence. Case reports have noted secondary SRCCs in *CDH1* mutation patients in the colon and appendix [16]. One study has noted an increased risk of colorectal cancer in a family with a *CDH1* missense germline mutation [77,78]. We have therefore used cases of sporadic gastric SRCCs in SEER to crudely estimate possible secondary cancer risks in our *CDH1* mutation patients, recognizing that SRCCs comprise 10–18% of all gastric cancer cases whereas HDGC are fewer at 1–3% [15]. However, our SEER results reproduced the increased lobular breast cancer risk already known in HDGC patients. This is also a particularly novel result as this would suggest that the mechanisms behind gastric SRCC, regardless of mutation status, might also confer an increased lobular breast cancer risk. This warrants further investigation. Our results also additionally uncover an increased risk of colorectal SRCC. The reciprocal risk of gastric SRCC among colorectal SRCC patients suggests that these two groups may share similar genetic risk factors, which could include *CDH1* and other related mutations. There is currently no consensus regarding the overall utility of increased colorectal cancer screening on the basis of *CDH1* mutation status alone [6]. If the link between colorectal SRCCs and *CDH1* mutations can be more directly established, high-risk endoscopic colorectal screening may be an important management strategy for these patients. Finally, the increased risk of sporadic secondary gastric and colorectal SRCCs is seen for lobular breast cancer patients over ductal breast cancer patients. These results overall can lead to a conjecture of an overarching signet-ring cell syndrome between gastric, colorectal, and lobular breast cancers (Figure 2).

The results of our study are not without limitations. Overall, the study of the management *CDH1*-mutation driven HDGC is fragmented to small case studies that are further undermined by an under appreciation that not all *CDH1* mutations carry an equivalent disease burden potential [79,80,81]. Our own cohort represents one of the largest known, but numbers only about 100 patients. Despite this, our results regarding endoscopic, pathologic, and secondary cancers are among the largest to date. As most studies involving *CDH1* mutation patients are either case reports or very small case series, there are no studies designed to directly compare endoscopic sampling techniques. This precludes conduct of a formalized meta-analysis. Nevertheless, we have demonstrated that the diagnostic yield of random biopsies in the literature is consistent with our results, and the estimate of the diagnostic yield from the Cambridge Protocol is likely a reasonable representation of the real-world reliability of this test. It is unlikely that the lack of improvement with Cambridge Protocol is due to publication bias, as we can demonstrate that that there are no significant differences in gender, age at surgery, cancer stage, or number of foci found on final pathology in the two groups.

We wish to explicitly acknowledge that our findings presented in Section 3.3 from the SEER database relate only to presumed sporadic cases of gastric, colorectal, and lobular breast cancer. Any applicability to HDGC is a postulation that requires further investigation from the amalgamation of patient outcomes across cohorts worldwide, given the rarity of this disease. A major limitation of SEER is that no genetic information is available in this database. Our motivation for conducting this aim of our study arose from our findings of secondary cancers in our *CDH1* cohort, and large databases like SEER serve as the only currently available patient data repositories to explore potential additional cancer risks among populations with rare cancers. As most of our patients have a single *CDH1* mutation [NM_004360.4(CDH1):c.2398delC (p.Arg800Alafs)], it is possible that our findings of secondary cancers will not translate across other *CDH1* germline mutations. However, the strength of our study is that we have among the largest *CDH1* germline mutation cohorts with up to nearly 20 years of follow-up data, which may explain our findings of other secondary cancers.

## 5. Conclusions

Overall, while patients are contemplating PTG, systematic endoscopic examination and biopsy protocols should be employed to rule out active early disease. However, clinicians must be explicit with patients that these exams have extremely poor performance rates and should not be used as a reassuring surrogate for delaying surgery in the context of negative biopsy results. In order to better counsel patients at risk of HDGC, *CDH1* mutation epidemiology must be more comprehensively characterized. Therefore, a centralized repository of patient outcomes is necessary, given the rarity of these mutations in the general population. Such data would provide both patients and clinicians with more effective data to manage risk in those delaying or refusing PTG, and for uncovering new challenges patients may face after their natural clinical course changes following PTG, including other secondary cancers.

## Figures and Tables

**Table 1:** Demographics of patients in Newfoundland and Labrador on basis of PTG.

Surgical Status	PTG	No PTG	*p*-Value
***n* (%)**	67 (69)	30 (31)	
**Gender (%)**			
Male	28 (42)	16 (53)	0.29
Female	39 (58)	14 (47)	
**Age at Genetic Testing (Years)**			
Mean (Range)	42.2 (18.1–64.3)	48.8 (17.4–90.0)	0.048
Median	42.6	51.1	
**Follow-up Time (Years)**			
Mean (Range)	10.5 (2.4–16.2)	7.5 (2.0–17.3)	0.003
Median	11.8	4.7	
***CDH1* (HGVS) Mutation (*n*) (%)**			0.17
NM_004360.4(CDH1):c.2398delC	59 (88)	28 (93)	
(p.Arg800Alafs)			
NM_004360.5(CDH1):c.1189A>T	2 (3)	0 (0)	
(p.Lys397Ter)			
NM_004360.5(CDH1):c.447_453	1 (1)	2 (7)	
CAGAAGA [1] (p.Gln152fs)			
Unknown	5 (7)	0 (0)	
**Endoscopy**			
No	7 (10)	6 (20)	0.20
Yes	60 (90)	24 (80)	
**Number of Scopes**			
Mean (Range)	2.7 (1–19)	5.9 (1–21)	0.002
Median	1	4	
**Surveillance Time (Years)**			
Mean (Range)	1.9 (0.1–10.3)	7.2 (2.0–17.3)	<0.001
Median	1.2	4.7	
**Prophylactic Mastectomy (*n*) (%)**			
Yes	17 (44)	0 (0)	0.003
No	22 (56)	14 (100)	
**Death (*n*) (%)**			
No	62 (93)	25 (83)	0.17
Yes	5 (7)	5 (17)	
Mean Age (Range)	59.9 (49.2–72.2)	78.1 (52.9–90.1)	0.07
Median	59.9	88.6	
Cause	Natural causes (×1), postoperative complications (×1), pancreatic adenoCa (×1), Breast lobular (SRCC) (×1), colon adenoCa (SRCC) (×1)	Natural causes (×3), melanoma (×1), rectal cancer (×1)	



HGVS (Human Genome Variation Society), adenoCa (adenocarcinoma).

**Table 2:** Overview of female *CDH1* mutation patients undergoing prophylactic mastectomy (16 out of 17 patients) in Newfoundland and Labrador.

Mastectomy Type	Prophylactic Bilateral Mastectomy	Completion Mastectomy	*p*-Value
***n***	9	7	
**Age (Years)**			0.007
Mean (Range)	42.3 (30.6–55.6)	53.6 (40.4–60.5)	
Median	41.3	55.2	
**Time from *CDH1* Genetic Testing (Years)**			0.06
Mean (Range)	6.2 (1.6–10.5)	3.3 (0.2–9.2)	
Median	5.9	2.5	
**Time from Initial Breast Cancer (Months)**	N/A		N/A
Mean (Range)		20.7 (1.4–57.3)	
Median		10.0	
**Findings (*n*) (%)**			N/A
Normal	1 (11)	2 (29)	
ALH	4 (44)	2 (29)	
ADH	-	1 (14)	
PASH	1 (11)	-	
LCIS	3 (33)	1 (14)	
DCIS	1 (11)	1 (14)	
Intraductal Papilloma	1 (11)	-	
Lobular Metaplasia	-	1 (14)	
Lobular Breast Cancer	-	1 (14)	

Prophylactic bilateral mastectomy refers to patients with no known breast cancer diagnosis who underwent removal of both breasts in the same procedure. Completion mastectomy refers to patients with a previous breast cancer diagnosis who then had subsequent surgery to remove the contralateral breast and/or any remaining ipsilateral breast tissue if the initial surgery was a lumpectomy. N/A (not applicable), ALH (atypical lobular hyperplasia), ADH (atypical ductal hyperplasia), PASH (pseudoangiomatous stromal hyperplasia), LCIS (lobular carcinoma in situ), DCIS (ductal carcinoma in situ).

**Table 3:** Overview of correlation of endoscopic biopsy results to final PTG specimen results in the Newfoundland and Labrador cohort.

Endoscopic Biopsy Result	Cancer Positive	Cancer Negative	*p*-Value
***n* (%)**	15	44	
**Cancer on Pathology (*n*) (%)**			0.28
**Yes**	14 (93)	36 (82)	
Tis	0 (0)	1 (2)	
T1a	13 (87)	35 (80)	
T2	1 (7)	0 (0)	
**No**	1 (7)	8 (18)	
**Gender (*n*) (%)**			0.70
Male	7 (47)	18 (41)	
Female	8 (53)	26 (59)	
***CDH1* (HGVS) Mutation (*n*) (%)**			0.49
NM_004360.4(CDH1):c.2398delC (p.Arg800Alafs)	14 (93)	38 (86)	
NM_004360.5(CDH1):c.1189A>T (p.Lys397Ter)	1 (7)	1 (2)	
NM_004360.5(CDH1):c.447_453 CAGAAGA [1] (p.Gln152fs)	0 (0)	1 (2)	
Unknown	0 (0)	4 (9)	
**Age at Genetic Testing (Years)**			0.003
Mean (Range)	34.0 (18.1–64.3)	44.3 (20.6–63.4)	
Median	32.3	46.8	
**Age at Surgery (Years)**			0.015
Mean (Range)	37.4 (21.7–72.6)	45.7 (22.9–63.7)	
Median	35.9	47.8	
**Number of Endoscopies (*n*)**			<0.001
Mean (Range)	5.4 (1–19)	1.8 (1–6)	
Median	3	1	
**Time from Genetic Testing to Surgery (Years)**			0.006
Mean (Range)	3.3 (0.1–10.3)	1.5 (0.1–9.1)	
Median	1.9	0.8	
**Time from Last Endoscopy to Surgery (Months)**			0.20
Mean (Range)	7.6 (2.25–19.2)	12.3 (0.7–59.8)	
Median	5.7	8.7	
**Number of Biopsies (Last Scope)**			0.02
Mean (Range)	16.9 (10–22)	12.2 (1–30)	
Median	18	13	
***Helicobacter Pylori* (*n*) (%)**			0.91
Yes	11 (73)	33 (75)	
No	1 (7)	4 (9)	
Unknown	3 (20)	7 (16)	
**Foci Count (*n*) (%)**			0.71
<3	6 (40)	20 (45)	
≥3	9 (60)	24 (55)	
**Embedding Protocol (Slides)**			0.07
**Total (*n*) (%)**	10 (67)	39 (89)	
Mean (Range)	53 (70–197)	147 (79–325)	
Median	99	128	
**Representative (*n*) (%)**	5 (33)	4 (9)	
Mean (Range)	29 (19–49)	40 (14–57)	
Median	27	45	
**Unknown (*n*) (%)**	0 (0)	1 (2)	

Tis (in situ disease).

**Table 4:** Pooled results from systematic review comparing random biopsies to Cambridge Protocol biopsies in the surveillance of asymptomatic *CDH1* mutation carriers.

Endoscopy Biopsy Protocol	Random	Cambridge	*p*-Value
***n***	154	112	
**Endoscopic Result** **(Positive Pathology) (*n*) (%)**			0.27
Negative	106 (79)	67 (73)	
Positive	28 (21)	25 (27)	
**Endoscopic Result** **(Negative Pathology) (*n*) (%)**			1.00
Negative	19 (95)	19 (95)	
Positive	1 (5)	1 (5)	
**Number of Biopsies (Last Scope)**		minimum 30	N/A
Mean (Range) (*n* = 66 for Random)	14.8 (1–60)		
Median	14.5		
**Gender (*n*) (%)**			0.81
Male	58	22	
Female	88	36	
Unknown	8	54	
**Age at Surgery (Years)**			0.37
Mean (Range) (*n* = 153/71)	40.4 (14–73)	42.0 (14–68)	
Median	41	41	
**Cancer on Pathology (*n*) (%)**			0.25
**Yes**	134 (87.0)	92 (82.0)	
Tis	4 (2.6)	3 (2.7)	
T1a	128 (83.1)	83 (74.1)	
T1b	1 (0.6)	2 (1.8)	
T2	1 (0.6)	1 (0.9)	
T3	0 (0)	3 (2.7)	
**No**	20 (13.0)	20 (17.9)	
**Foci Count (*n*) (%)**			0.47
0	21 (13.6)	21 (18.8)	
1–2	26 (16.9)	13 (11.6)	
3–10	30 (19.5)	20 (17.9)	
11–50	29 (18.8)	21 (18.8)	
51–100	3 (1.9)	4 (3.6)	
>101	5 (3.2)	1 (0.9)	
Unknown	40 (26.0)	32 (28.6)	
**Embedding Protocol (*n*) (%)**			0.20
Total	114 (74)	74 (66)	
Representative	12 (8)	16 (14)	
Unknown	28 (18)	22 (20)	

N/A, not applicable.

**Table 5:** Estimated hazard risk ratios (HR) with 95% confidence intervals (CI) for secondary cancers among patients with a primary sporadic gastric SRCC compared to other patients with any other gastric cancer type and conventional gastric adenocarcinoma from SEER.

Secondary Cancers following Primary Gastric Cancer	Gastric SRCC vs. Any Gastric Cancer HR (95% CI)	Gastric SRCC vs. Conventional Gastric Adenocarcinoma HR (95% CI)
Any Cancer (32,056)	0.96 (0.93–0.99)	**1.05 (1.01–1.09)**
Male (20,686)	0.94 (0.89–0.99)	1.01 (0.96–1.06)
Female (11,370)	0.95 (0.90–1.00)	1.06 (1.00–1.12)
Any SRCC (115)	**3.38 (2.25–5.06)**	**3.84 (2.48–5.96)**
Male (76)	**2.71 (1.59–4.62)**	**2.95 (1.68–5.19)**
Female (39)	**4.68 (2.33–9.40)**	**6.27 (2.68–14.7)**
Any Breast Cancer (Female) (3656)	**1.18 (1.08–1.29)**	**1.25 (1.13–1.37)**
Lobular Breast Cancer (Female) (801)	**1.68 (1.41–2.00)**	**1.72 (1.43–2.07)**
Ductal Breast Cancer (Female) (2353)	1.04 (0.93–1.17)	1.12 (0.99–1.26)
Any Colorectal Cancer (5304)	0.94 (0.86–1.03)	0.99 (0.90–1.08)
Male (3358)	0.99 (0.88–1.11)	1.03 (0.91–1.16)
Female (1946)	0.86 (0.75–0.99)	0.91 (0.78–1.05)
Colorectal SRCC (52)	**3.27 (1.81–5.91)**	**3.90 (2.04–7.44)**
Male (30)	**2.97 (1.30–6.79)**	**3.28 (1.37–7.83)**
Female (22)	**3.56 (1.46–8.65)**	**4.87 (1.64–14.4)**
Conventional Colorectal		
Adenocarcinoma (3077)	0.89 (0.79–1.01)	0.91 (0.80–1.04)
Male (1934)	0.94 (0.81–1.10)	0.96 (0.82–1.13)
Female (1143)	0.81 (0.67–1.04)	0.82 (0.68–1.01)

There was a total of 172,375 cases of primary gastric cancer, of which 24,226 were sporadic (assumed) gastric SRCCs and 109,397 were conventional gastric adenocarcinomas. Total numbers of patients (*n*) within each secondary cancer category are indicated. All results had a competing risk adjustment for death from primary gastric cancer, and were both age and gender adjusted. Bolded results indicate *p* < 0.05.

**Table 6:** Estimated hazard risk ratios (HR) with 95% confidence intervals (CI) for secondary cancers among patients with a primary sporadic colorectal SRCC compared to other patients with any other colorectal cancer type and conventional colorectal adenocarcinoma from SEER.

Secondary Cancers following Primary Colorectal Cancer	Colorectal SRCC vs. Any Colorectal Cancer HR (95% CI)	Colorectal SRCC vs. Conventional Colorectal Adenocarcinoma HR (95% CI)
Any Cancer (227,305)	0.97 (0.92–1.02)	1.06 (1.01–1.12)
Male (127,582)	0.93 (0.86–1.00)	1.03 (0.96–1.10)
Female (99,723)	1.04 (0.96–1.02)	**1.12 (1.03–1.21)**
Any SRCC (797)	**6.61 (4.71–9.27)**	**7.91 (5.61–11.2)**
Male (462)	**5.82 (3.63–9.32)**	**6.99 (4.34–11.3)**
Female (335)	**7.79 (4.79–12.7)**	**9.21 (5.61–15.1)**
Any Breast Cancer (Female) (35,515)	0.99 (0.87–1.13)	1.05 (0.93–1.20)
Lobular Breast Cancer (Female) (6576)	1.23 (0.94–1.61)	**1.38 (1.05–1.81)**
Ductal Breast Cancer (Female) (24,314)	0.91 (0.77–1.07)	0.95 (0.81–1.12)
Any Gastric Cancer (5210)	**1.47 (1.12–1.95)**	**1.60 (1.21–2.12)**
Male (3294)	1.31 (0.91–1.91)	1.45 (1.00–2.11)
Female (1916)	**1.75 (1.15–2.67)**	**1.87 (1.23–2.85)**
Gastric SRCC (574)	**4.67 (2.93–7.46)**	**5.47 (3.41–8.77)**
Male (328)	**3.58 (1.77–7.22)**	**4.23 (2.09–8.57)**
Female (246)	**6.24 (3.32–11.7)**	**7.17 (3.79–13.6)**
Conventional Gastric Adenocarcinoma (3336)	0.97 (0.63–1.49)	1.04 (0.67–1.59)
Male (2223)	1.05 (0.63–1.75)	1.13 (0.68–1.89)
Female (1113)	0.82 (0.37–1.83)	0.82 (0.38–1.90)

There was a total of 1,068,086 cases of primary colorectal cancer, of which 9254 were sporadic (assumed) colorectal SRCCs and 666,362 were conventional colorectal adenocarcinomas. Total number of patients (*n*) within each secondary cancer category are indicated. All results had a competing risk adjustment for death from primary colorectal cancer, and were both age and gender adjusted. Bolded results indicate *p* < 0.05.

**Table 7:** Estimated hazard risk ratios (HR) with 95% confidence intervals (CI) for secondary cancers among patients with a primary sporadic lobular breast cancer compared to other patients with any other breast cancer type and ductal breast cancer in SEER.

Secondary Cancers following Primary Lobular Breast Cancer	Lobular Breast Cancer vs. Any Breast Cancer HR (95% CI)	Lobular Breast Cancer vs. Ductal Breast Cancer HR (95% CI)
Any Cancer (235,984)	**1.14 (1.13–1.15)**	**1.12 (1.11–1.13)**
Any SRCC (1140)	**1.73 (1.52–1.97)**	**1.78 (1.56–2.04)**
Any Gastric Cancer (3612)	**1.28 (1.18–1.39)**	**1.29 (1.19–1.40)**
Gastric SRCC (667)	**1.97 (1.67–2.32)**	**2.00 (1.68–2.34)**
Conventional Gastric Adenocarcinoma (1950)	**1.16 (1.03–1.29)**	**1.17 (1.05–1.32)**
Any Colorectal Cancer (35,343)	**1.06 (1.03–1.09)**	**1.04 (1.01–1.07)**
Colorectal SRCC (258)	**1.34 (1.01–1.80)**	**1.42 (1.05–1.91)**
Conventional Colorectal Adenocarcinoma (20,993)	1.00 (0.96–1.03)	0.97 (0.94–1.01)

There was a total of 1,703,071 cases of primary breast cancer in females, of which 340,930 were sporadic (assumed) lobular breast cancers and 1,157,464 were ductal breast cancers. Total number of patients (*n*) within each secondary cancer category are indicated. All results had a competing risk adjustment for death from primary breast cancer, and were age adjusted. Bolded results indicate *p* < 0.05.

## Data Availability

All raw data for our systematic review is presented in the Supplementary File with references to all sources. Raw data from SEER (www.seer.cancer.gov (accessed on 19 April 2021)) is publicly available via signed agreements.

## Supplementary Materials

The following are available online at https://www.mdpi.com/article/10.3390/cancers13112622/s1, Table S1: Systematic review search strings, Table S2: Inclusion and exclusion criteria for systematic review study selection. Table S3: Risk of bias assessment for all included case series and case reports. Table S4: Data extraction table from included studies. Table S5: Variable definition in SEER secondary cancer analysis. Table S6: Exclusion criteria and counts of all cases in SEER secondary cancer analysis. Table S7: Secondary cancers observed in Newfoundland and Labrador *CDH1* mutation cohort.

## References

[1] Fitzgerald R.C., Hardwick R., Huntsman D., Carneiro F., Guilford P., Blair V., Chung D.C., Norton J., Ragunath K., Van Krieken J.H. (2010). Hereditary diffuse gastric cancer: Updated consensus guidelines for clinical management and directions for future research. J. Med. Genet..

[2] Becker K.F., Atkinson M., Reich U., Becker I., Nekarda H., Siewert J.R., Höfler H. (1994). E-cadherin gene mutations provide clues to diffuse type gastric carcinomas. Cancer Res..

[3] Hansford S., Kaurah P., Li-Chang H., Woo M., Senz J., Pinheiro H., Schrader K.A., Schaeffer D.F., Shumansky K., Zogopoulos G. (2015). Hereditary diffuse gastric cancer syndrome: Cdh1 mutations and beyond. JAMA Oncol..

[4] Guilford P., Hopkins J., Harraway J., McLeod M., McLeod N., Harawira P., Taite H., Scoular R., Miller A.C., Reeve A.E. (1998). E-cadherin germline mutations in familial gastric cancer. Nat. Cell Biol..

[5] Dossus L., Benusiglio P.R. (2015). Lobular breast cancer: Incidence and genetic and non-genetic risk factors. Breast Cancer Res..

[6] Van Der Post R.S., Vogelaar I.P., Carneiro F., Guilford P., Huntsman D., Hoogerbrugge N., Caldas C., Schreiber K.E.C., Hardwick R.H., Ausems M.G.E.M. (2015). Hereditary diffuse gastric cancer: Updated clinical guidelines with an emphasis on germlineCDH1mutation carriers. J. Med. Genet..

[7] van der Post R.S., Oliveira C., Guilford P., Carneiro F. (2019). Hereditary gastric cancer: What’s new? Update 2013–2018. Fam. Cancer.

[8] Blair V.R., McLeod M., Carneiro F., Coit D.G., D’Addario J.L., van Dieren J.M., Harris K.L., Hoogerbrugge N., Oliveira C., van der Post R.S. (2020). Hereditary diffuse gastric cancer: Updated clinical practice guidelines. Lancet Oncol..

[9] Majewski I.J., Kluijt I., Cats A., Scerri T.S., De Jong D., Kluin R.J.C., Hansford S., Hogervorst F.B.L., Bosma A.J., Hofland I. (2013). An α-E-catenin (CTNNA1) mutation in hereditary diffuse gastric cancer. J. Pathol..

[10] Shenoy S. (2019). CDH1 (E-Cadherin) Mutation and Gastric Cancer: Genetics, Molecular Mechanisms and Guidelines for Management. Cancer Manag. Res..

[11] Melo S., Figueiredo J., Fernandes M.S., Gonçalves M., Morais-De-Sá E., Sanches J.M., Seruca R. (2017). Predicting the Functional Impact of CDH1 Missense Mutations in Hereditary Diffuse Gastric Cancer. Int. J. Mol. Sci..

[12] Huynh J.M., Laukaitis C.M. (2016). Panel testing reveals nonsense and missense CDH 1 mutations in families without hereditary diffuse gastric cancer. Mol. Genet. Genom. Med..

[13] Figueiredo J., Melo S., Carneiro P., Moreira A.M., Fernandes M.S., Ribeiro A.S., Guilford P., Paredes J., Seruca R. (2019). Clinical spectrum and pleiotropic nature of *cdh1* germline mutations. J. Med. Genet..

[14] Oliveira C., Sousa S., Pinheiro H., Karam R., Bordeira–Carriço R., Senz J., Kaurah P., Carvalho J., Pereira R., Gusmão L. (2009). Quantification of Epigenetic and Genetic 2nd Hits in CDH1 During Hereditary Diffuse Gastric Cancer Syndrome Progression. Gastroenterology.

[15] Benesch M.G., Mathieson A. (2020). Epidemiology of Signet Ring Cell Adenocarcinomas. Cancers.

[16] Hamilton L.E., Jones K., Church N., Medlicott S. (2013). Synchronous appendiceal and intramucosal gastric signet ring cell carcinomas in an individual with CDH1-associated hereditary diffuse gastric carcinoma: A case report of a novel association and review of the literature. BMC Gastroenterol..

[17] Richards F.M., McKee S.A., Rajpar M.H., Cole T.R.P., Evans D.G.R., Jankowski J., McKeown C., Sanders D.S.A., Maher E.R. (1999). Germline E-cadherin Gene (CDH1) Mutations Predispose to Familial Gastric Cancer and Colorectal Cancer. Hum. Mol. Genet..

[18] Ottenhof N.A., De Wilde R.F., Morsink F.H., De Leng W.W., Ausems M.G., Morreau H., Van Hillegersberg R., Offerhaus G.J.A., Milne A.N. (2012). Pancreatic ductal adenocarcinoma in hereditary diffuse gastric cancer. A case report. Hum. Pathol..

[19] Roberts M.E., Ranola J.M.O., Marshall M.L., Susswein L.R., Graceffo S., Bohnert K., Tsai G., Klein R.T., Hruska K.S., Shirts B.H. (2019). Comparison of CDH1 Penetrance Estimates in Clinically Ascertained Families vs Families Ascertained for Multiple Gastric Cancers. JAMA Oncol..

[20] Laszkowska M., Silver E.R., Schrope B., Kastrinos F., Wang T.C., Hur C. (2020). Optimal Timing of Total Gastrectomy to Prevent Diffuse Gastric Cancer in Individuals With Pathogenic Variants in CDH1. Clin. Gastroenterol. Hepatol..

[21] Muir J., Aronson M., Esplen M.-J., Pollett A., Swallow C.J. (2016). Prophylactic Total Gastrectomy: A Prospective Cohort Study of Long-Term Impact on Quality of Life. J. Gastrointest. Surg..

[22] Kaurah P., Talhouk A., Macmillan A., Lewis I., Chelcun-Schreiber K., Yoon S.S., Huntsman D. (2019). Hereditary diffuse gastric cancer: Cancer risk and the personal cost of preventive surgery. Fam. Cancer.

[23] Strong V.E., Gholami S., Shah M.A., Tang L.H., Janjigian Y.Y., Schattner M., Selby L.V., Yoon S.S., Salo-Mullen E., Stadler Z.K. (2017). Total gastrectomy for hereditary diffuse gastric cancer at a single center: Postsurgical outcomes in 41 patients. Ann. Surg..

[24] Rocha J.P., Gullo I., Wen X., Devezas V., Baptista M., Oliveira C., Carneiro F. (2018). Pathological features of total gastrectomy specimens from asymptomatic hereditary diffuse gastric cancer patients and implications for clinical management. Histopathology.

[25] Hüneburg R., Marwitz T., Van Heteren P., Weismüller T.J., Trebicka J., Adam R., Aretz S., Bouza A.P., Pantelis D., Kalff J.C. (2016). Chromoendoscopy in combination with random biopsies does not improve detection of gastric cancer foci in CDH1 mutation positive patients. Endosc. Int. Open.

[26] Kumar S., Katona B., Long J.M., Domchek S., Rustgi A.K., Roses R., Ginsberg G.G. (2020). Endoscopic Ultrasound Has Limited Utility in Diagnosis of Gastric Cancer in Carriers of CDH1 Mutations. Clin. Gastroenterol. Hepatol..

[27] Hebbard P.C., MacMillan A., Huntsman D., Kaurah P., Carneiro F., Wen X., Kwan A., Boone D., Bursey F., Green J. (2009). Prophylactic Total Gastrectomy (PTG) for Hereditary Diffuse Gastric Cancer (HDGC): The Newfoundland Experience with 23 Patients. Ann. Surg. Oncol..

[28] Moga C., Guo B., Schopflocher D., Harstall C. (2012). Development of a Quality Appraisal Tool for Case Series Studies Using a Modified Delphi Technique.

[29] Moola S., Munn Z., Tufanaru C., Aromataris E., Sears K., Sfetcu R., Currie M., Qureshi R., Mattis P., Lisy K., Aromataris E., Munn Z. (2020). Chapter 7: Systematic reviews of etiology and risk. JBI Manual for Evidence Synthesis.

[30] Surveillance, Epidemiology, and End Results (Seer) Program (www.Seer.Cancer.Gov) Research Data (1975-2016), National Cancer Institute, DCCPS, Surveillance Research Program, Released April 2019, Based on the November 2018 Submission. www.Seer.Cancer.Gov.

[31] Fine J.P., Gray R.J. (1999). A proportional hazards model for the subdistribution of a competing risk. J. Am. Stat. Assoc..

[32] Aziz M., Madan R., Bansal A. (2018). Hereditary Diffuse Gastric Cancer: More than What Meets the Endoscopic Eye. Kans. J. Med..

[33] Barber M., Save V., Carneiro F., Dwerryhouse S., Lao-Sirieix P., Hardwick R., Caldas C., Fitzgerald R. (2008). Histopathological and molecular analysis of gastrectomy specimens from hereditary diffuse gastric cancer patients has implications for endoscopic surveillance of individuals at risk. J. Pathol..

[34] Bardram L., Hansen T.V.O., Gerdes A.-M., Timshel S., Friis-Hansen L., Federspiel B. (2014). Prophylactic total gastrectomy in hereditary diffuse gastric cancer: Identification of two novel CDH1 gene mutations—A clinical observational study. Fam. Cancer.

[35] Black M.D., Kaneshiro R., I Lai J., Shimizu D.M. (2014). Hereditary Diffuse Gastric Cancer Associated with E-cadherin Germline Mutation: A Case Report. Hawaii J. Med. Public Health J. Asia Pac. Med. Public Health.

[36] Caron O., Schielke A., Svrcek M., Fléjou J.-F., Garzon J., Olschwang S., Sézeur A. (2008). Usefulness of Prophylactic Gastrectomy in a Novel Large Hereditary Diffuse Gastric Cancer (HDGC) Family. Am. J. Gastroenterol..

[37] Castro R., Lobo J., Pita I., Videira F., Pedro-Afonso L., Dinis-Ribeiro M., Brandão C. (2020). Random biopsies in patients harboring a cdh1 mutation: Time to change the approach?. Rev. Esp. Enferm. Dig..

[38] Charlton A., Blair V., Shaw D., Parry S., Guilford P., Martin I.G. (2004). Hereditary diffuse gastric cancer: Predominance of multiple foci of signet ring cell carcinoma in distal stomach and transitional zone. Gut.

[39] Chen Y., Kingham K., Ford J.M., Rosing J., Van Dam J., Jeffrey R.B., Longacre T.A., Chun N., Kurian A., Norton J.A. (2011). A Prospective Study of Total Gastrectomy for CDH1-Positive Hereditary Diffuse Gastric Cancer. Ann. Surg. Oncol..

[40] Chun Y.S., Lindor N.M., Smyrk T.C., Petersen B.T., Burgart L.J., Guilford P.J., Donohue J.H. (2001). Germline e-cadherin gene mutations: Is prophylactic total gastrectomy indicated?. Cancer.

[41] Devezas V., Baptista M., Gullo I., Rocha J., Sousa F., Xiaogang W., Preto J., Costa S., Castedo S., Garrido L. (2020). Risk-reducing total gastrectomy in asymptomatic cdh1 carriers: Experience of a tertiary hospital. Eur. Surg..

[42] DiBrito S.R., Blair A.B., Prasath V., Habibi M., Harmon J.W., Duncan M.D. (2020). Total Gastrectomy for CDH-1 Mutation Carriers: An Institutional Experience. J. Surg. Res..

[43] Francis W.P., Rodrigues D.M., Perez N.E., Lonardo F., Weaver N., Webber J.D. (2007). Prophylactic laparoscopic-assisted total gastrectomy for hereditary diffuse gastric cancer. JSLS J. Soc. Laparoendosc. Surg..

[44] Frebourg T., Oliveira C., Hochain P., Karam R., Manouvrier S., Graziadio C., Vekemans M., Hartmann A., Baert-Desurmont S., Alexandre C. (2005). Cleft lip/palate and CDH1/E-cadherin mutations in families with hereditary diffuse gastric cancer. J. Med Genet..

[45] Friedman M., Adar T., Patel D., Lauwers G.Y., Yoon S.S., Mullen J.T., Chung D.C. (2021). Surveillance Endoscopy in the Management of Hereditary Diffuse Gastric Cancer Syndrome. Clin. Gastroenterol. Hepatol..

[46] Fujita H., Lennerz J.K., Chung D.C., Patel D., Deshpande V., Yoon S.S., Lauwers G.Y. (2012). Endoscopic surveillance of patients with hereditary diffuse gastric cancer: Biopsy recommendations after topographic distribution of cancer foci in a series of 10 cdh1-mutated gastrectomies. Am. J. Surg. Pathol..

[47] Gjyshi O., Vashi P., Seewald L., Kohan M., Abboud E., Fowler E., Suppiah R., Halabi H. (2018). Therapeutic and prophylactic gastrectomy in a family with hereditary diffuse gastric cancer secondary to a CDH1 mutation: A case series. World J. Surg. Oncol..

[48] Hackenson D., Edelman D.A., McGuire T., Weaver D.W., Webber J.D. (2010). Prophylactic Laparoscopic Gastrectomy for Hereditary Diffuse Gastric Cancer: A Case Series in a Single Family. JSLS J. Soc. Laparoendosc. Surg..

[49] Herráiz M., Valenti V., Sola J., Pérez-Rojo P., Rotellar F., Cienfuegos J.A. (2012). Hereditary diffuse gastric cancer: Strategies to reduce tumoral risk. Rev. Española Enferm. Dig..

[50] Huntsman D.G., Carneiro F., Lewis F.R., MacLeod P.M., Hayashi A., Monaghan K.G., Maung R., Seruca R., Jackson C.E., Caldas C. (2001). Early Gastric Cancer in Young, Asymptomatic Carriers of Germ-Line E-Cadherin Mutations. N. Engl. J. Med..

[51] Jacobs M.F., Dust H., Koeppe E., Wong S., Mulholland M., Choi E.-Y., Appelman H., Stoffel E.M. (2019). Outcomes of Endoscopic Surveillance in Individuals With Genetic Predisposition to Hereditary Diffuse Gastric Cancer. Gastroenterology.

[52] Jadot V., Segers K., Bours V., Kohnen L., Honoré P., Martin M., De Flines J., Mutijima E., Leclercq P. (2019). Hereditary diffuse gastric cancer: Case serie of 8 patients from a single family and literature review. Rev. Med. Liege.

[53] Khare M., Weaver D.W., Hart J.L. (2011). Case series of prophylactic laparoscopic total gastrectomy for hereditary diffuse gastric cancer with cadherin gene mutation. Surg. Endosc. Other Interv. Tech..

[54] Li J., McBean E., Li X., Berho M., Szomstein S., Rosenthal R.J. (2013). Laparoscopic Prophylactic Total Gastrectomy With Linear Stapler Side-to-Side Esophagojejunal Anastomosis for Hereditary Diffuse Gastric Cancer Syndrome in 2 Siblings. Surg. Laparosc. Endosc. Percutaneous Tech..

[55] Moslim M.A., Heald B., Tu C., Burke C.A., Walsh R.M. (2018). Early genetic counseling and detection of CDH1 mutation in asymptomatic carriers improves survival in hereditary diffuse gastric cancer. J. Surg. Educ..

[56] Ruiz V.M., Jimeno P., De Angulo D.R., Ortiz Á., De Haro L.F.M., Marín M., Cascales P., García G.R., Ruiz E.O., Parrilla P. (2018). Is prophylactic gastrectomy indicated for healthy carriers of CDH1 gene mutations associated with hereditary diffuse gastric cancer?. Rev. Española Enferm. Dig..

[57] Oelschlager B.K., Yigit T., Kaufman J.A., Pellegrini C.A. (2005). Hereditary diffuse gastric cancer. MedGenMed.

[58] Pandalai P., Lauwers G.Y., Chung D.C., Patel D., Yoon S.S. (2011). Prophylactic total gastrectomy for individuals with germline CDH1 mutation. Surgery.

[59] Pantelis D., Hüneburg R., Adam R., Holzapfel S., Gevensleben H., Nattermann J., Strassburg C.P., Aretz S., Kalff J.C. (2016). Prophylactic total gastrectomy in the management of hereditary tumor syndromes. Int. J. Color. Dis..

[60] Shepard B., Yoder L., Holmes C. (2016). Prophylactic Total Gastrectomy for Hereditary Diffuse Gastric Cancer. ACG Case Rep. J..

[61] Svrcek M. (2011). [case n(o) 6: Signet ring cell intramucosal carcinoma in hereditary diffuse gastric cancer with mutated cdh1 gene]. Ann. Pathol..

[62] Van Dieren J.M., Kodach L.L., Hartog P.D., Van Der Kolk L.E., Sikorska K., Van Velthuysen M.-L.F., Van Sandick J.W., Koemans W.J., Snaebjornsson P., Cats A. (2020). Gastroscopic surveillance with targeted biopsies compared with random biopsies in CDH1 mutation carriers. Endoscopy.

[63] Van Kouwen M.C.A., Drenth J.P.H., Oyen W.J., De Bruin J.H.F.M., Ligtenberg M.J., Bonenkamp J.J.H., Van Krieken J.H.J.M., Nagengast F.M. (2004). [18F]Fluoro-2-deoxy-d-glucose Positron Emission Tomography Detects Gastric Carcinoma in an Early Stage in an Asymptomatic E-Cadherin Mutation Carrier. Clin. Cancer Res..

[64] Wickremeratne T., Lee C.H., Kirk J., Charlton A., Thomas G., Gaskin K.J. (2014). Prophylactic gastrectomy in a 16-year-old. Eur. J. Gastroenterol. Hepatol..

[65] Wilcox R., Perpich M., Noffsinger A., Posner M.C., Cooper K. (2011). Hereditary Diffuse Gastric Cancer: Multidisciplinary Case Report with Review of the Literature. Pathol. Res. Int..

[66] Kumar S., Long J.M., Ginsberg G.G., Katona B. (2019). Role of endoscopy in the management of hereditary diffuse gastric cancer syndrome. World J. Gastroenterol..

[67] Mi E.Z., Mi E.Z., di Pietro M., O’Donovan M., Hardwick R.H., Richardson S., Ziauddeen H., Fletcher P.C., Caldas C., Tischkowitz M. (2018). Comparative study of endoscopic surveillance in hereditary diffuse gastric cancer according to CDH1 mutation status. Gastrointest. Endosc..

[68] Hallowell N., Badger S., Richardson S., Caldas C., Hardwick R.H., Fitzgerald R.C., Lawton J. (2016). An investigation of the factors effecting high-risk individuals’ decision-making about prophylactic total gastrectomy and surveillance for hereditary diffuse gastric cancer (HDGC). Fam. Cancer.

[69] Hamilton J.G., Long J.M., Brandt A.C., Brower J., Symecko H., Salo-Mullen E.E., Christian S.N., Harstad T., Couch F.J., Garber J.E. (2019). Patients’ Medical and Psychosocial Experiences After Detection of a CDH1 Variant With Multigene Panel Testing. JCO Precis. Oncol..

[70] Hallowell N., Lawton J., Badger S., Richardson S., Hardwick R.H., Caldas C., Fitzgerald R.C. (2016). The Psychosocial Impact of Undergoing Prophylactic Total Gastrectomy (PTG) to Manage the Risk of Hereditary Diffuse Gastric Cancer (HDGC). J. Genet. Couns..

[71] Zhang H., Feng M., Feng Y., Bu Z., Li Z., Jia S., Ji J. (2018). Germline mutations in hereditary diffuse gastric cancer. Chin. J. Cancer Res..

[72] Tan R.Y.C., Ngeow J. (2015). Hereditary diffuse gastric cancer: What the clinician should know. World J. Gastrointest. Oncol..

[73] Corso G., Figueiredo J., Biffi R., Trentin C., Bonanni B., Feroce I., Serrano D., Cassano E., Annibale B., Melo S. (2014). E-cadherin germline mutation carriers: Clinical management and genetic implications. Cancer Metastasis Rev..

[74] Katona B.W., Clark D.F., Domchek S.M. (2019). CDH1 on Multigene Panel Testing: Look Before You Leap. J. Natl. Cancer Inst..

[75] Vos E.L., Salo-Mullen E.E., Tang L.H., Schattner M., Yoon S.S., Gerdes H., Markowitz A.J., Mandelker D., Janjigian Y., Offitt K. (2020). Indications for Total Gastrectomy in CDH1 Mutation Carriers and Outcomes of Risk-Reducing Minimally Invasive and Open Gastrectomies. JAMA Surg..

[76] Benusiglio P.R., Malka D., Rouleau E., De Pauw A., Buecher B., Noguès C., Fourme E., Colas C., Coulet F., Warcoin M. (2013). CDH1germline mutations and the hereditary diffuse gastric and lobular breast cancer syndrome: A multicentre study. J. Med Genet..

[77] Salahshor S., Hou H., Diep C., Loukola A., Zhang H., Liu T., Chen J., Iselius L., Rubio C., Lothe R. (2001). A germline E-cadherin mutation in a family with gastric and colon cancer. Int. J. Mol. Med..

[78] Luo W., Fedda F., Lynch P., Tan D. (2018). CDH1 Gene and Hereditary Diffuse Gastric Cancer Syndrome: Molecular and Histological Alterations and Implications for Diagnosis and Treatment. Front. Pharmacol..

[79] Lee H.E., Smyrk T.C., Zhang L. (2018). Histologic and immunohistochemical differences between hereditary and sporadic diffuse gastric carcinoma. Hum. Pathol..

[80] Pernot S., Voron T., Perkins G., Lagorce-Pages C., Berger A., Taieb J. (2015). Signet-ring cell carcinoma of the stomach: Impact on prognosis and specific therapeutic challenge. World J. Gastroenterol..

[81] Seevaratnam R., Coburn N., Cardoso R., Dixon M., Bocicariu A., Helyer L. (2011). A systematic review of the indications for genetic testing and prophylactic gastrectomy among patients with hereditary diffuse gastric cancer. Gastric Cancer.

