# Supplementary material for: CDH1 Gene Mutation Hereditary Diffuse Gastric Cancer Outcomes: Analysis of a Large Cohort, Systematic Review of Endoscopic Surveillance, and Secondary Cancer Risk Postulation
Source: Cancers (Basel). 2021 May 26;13(11):2622. doi: 10.3390/cancers13112622 (PMC8199234; doi:10.3390/cancers13112622)
Supplement: Supplementary file 1 [file cancers-13-02622-s001.zip › cancers-1207957-supplementary.pdf]

MEDLINE/Pubmed
("Stomach Neoplasms"[Mesh:NoExp] OR gastric[tiab]) AND ("Carcinoma, Signet Ring Cell"[Mesh:NoExp] OR signet[tiab] OR SRCC[tiab] OR CDH1[tiab]) AND ("Endoscopy, Digestive System"[Mesh] OR endoscop*[tiab]) AND gastrec*[tiab]

Embase
('stomach cancer'/exp OR 'gastric':ti,ab) ('signet':ti,ab OR 'srcc':ti,ab OR 'cdh1':ti,ab) AND 'endoscop*':ti,ab AND 'gastrec*':ti,ab

Table S1: Systematic review search strings.

Inclusion criteria:

- Participants with genetically verified *CDH1* mutation, and/or part of a known *CDH1* family with hereditary diffuse gastric cancer (meeting clinical criteria).
- Clinically asymptomatic.
- Undergone subsequent total prophylactic gastrectomy.

Exclusion criteria:

- Upper endoscopy not for *CDH1* surveillance purposes.
- Absence of gastric biopsy.
- Previous gastric cancer diagnosis.

Table S2: Inclusion and exclusion criteria for systematic review study selection. Full study design available at https://www.crd.york.ac.uk/prospero/display_record.php?RecordID=184631 (PROSPERO, CRD42020184631.

Risk of Bias Assessment for Included Case Series (≥3 patients)

			Barber et al, 2008	Bardram et al, 2014	Caron et al, 2008	Castro et al, 2020	Charlton et al, 2004	Chen et al, 2011	Chun et al, 2001	Devezas et al, 2020	DiBrito et al, 2020	Friedman et al, 2020	Fujita et al, 2012	Hackenson et al, 2010	Huneburg, et al 2016	Huntsman et al, 2001	Jacobs et al, 2019	Jadot et al, 2019	Khare et al, 2011	Kumar et al, 2020	Moslim et al, 2018	Munitiz Ruiz et al, 2019	Pandalai et al, 2011	van Dieren et al, 2020
**Study objective**																								
1	**Was the hypothesis/aim/objective of the study clearly stated?**	Yes (2) Partial (1) No (0)	2	2	1	2	2	2	1	2	2	2	2	2	2	1	2	2	2	2	2	2	2	2
**Study design**																								
2	**Was the study conducted prospectively?**	Yes (2) Unclear/No (0)	0	2	0	2	0	2	0	0	0	0	0	0	2	0	0	0	0	0	0	0	0	0
3	**Were the cases collected in more than one centre?**	Yes (2) Unclear/No (0)	0	0	0	0	0	0	0	0	0	0	0	0	0	0	0	0	0	0	0	0	0	0
4	**Were patients recruited consectively?**	Yes (2) Unclear/No (0)	0	2	0	0	0	2	0	2	0	2	2	0	0	0	0	2	0	0	0	0	2	0
**Study population**																								
5	**Were the characteristics of the patients included in the study described?**	Yes (2) Partial (1) No (0)	2	2	2	2	2	2	2	2	2	2	2	2	2	2	2	2	2	2	2	2	2	2
6	**Were the eligibility crieria (inclusion/exclusion) for entry into the study clearly stated?**	Yes (2) Partial (1) No (0)	2	2	2	2	1	2	1	2	2	2	2	2	2	2	2	2	1	2	2	2	2	2
7	**Did patients enter the study at a similar point in the disease?**	Yes (2) Unclear/No (0)	2	2	2	2	2	0	2	0	2	2	2	2	2	2	2	2	2	2	0	2	2	2
**Intervention and co-intervention**																								
8	**Was the intervention of interest clearly described?**	Yes (2) Partial (1) No (0)	2	2	2	2	2	2	2	2	2	1	2	2	2	1	2	2	2	2	1	1	1	2
9	**Were additional interventions (co-interventions) clearly described?**	Yes (2) Partial (1) No (0)	N/A	2	N/A	N/A	N/A	N/A	N/A	N/A	N/A	N/A	N/A	N/A	N/A	N/A	N/A	2	N/A	2	N/A	N/A	N/A	N/A
**Outcome measure**																								
10	**Were relevant outcome measures established a priori?**	Yes (2) Partial (1) No (0)	2	2	2	2	2	2	2	2	2	2	2	2	2	2	2	2	2	2	2	2	2	2
11	**Were outcome assessors blinded to the intervention that patients received?**	Yes (2) Unclear/No (0)	0	0	0	0	0	0	0	0	0	0	0	0	0	0	0	0	0	0	0	0	0	0
12	**Were the relevant outcomes measured using appropriate objective/subjective methods?**	Yes (2) Partial (1) No (0)	2	2	1	2	2	1	1	2	1	2	2	1	2	1	2	1	1	1	1	1	1	2
13	**Were the relevant outcome measures made before and after the intervention?**	Yes (2) Unclear/No (0)	N/A	N/A	N/A	N/A	N/A	N/A	N/A	N/A	N/A	N/A	N/A	N/A	N/A	N/A	N/A	N/A	N/A	N/A	N/A	N/A	N/A	N/A
**Statistical analysis**																								
14	**Were the statistical tests used to assess the relevant outcomes appropriate?**	Yes (2) Unclear/No (0)	2	0	0	2	0	2	0	2	2	2	2	0	2	0	2	2	2	2	2	2	2	2
**Results and conclusions**																								
15	**Was follow-up long enough for important events and outcomes to occur?**	Yes (2) Unclear/No (0)	N/A	N/A	N/A	N/A	N/A	N/A	N/A	N/A	N/A	N/A	N/A	N/A	N/A	N/A	N/A	N/A	N/A	N/A	N/A	N/A	N/A	N/A
16	**Were losses to follow-up reported?**	Yes (2) Unclear/No (0)	2	0	2	2	2	2	0	2	0	2	2	2	2	0	2	2	0	2	2	0	2	2
17	**Did the study provide estimates of random variablity in the data analysis of relevant outcomes?**	Yes (2) Partial (1) No (0)	N/A	N/A	N/A	N/A	N/A	N/A	N/A	N/A	N/A	N/A	N/A	N/A	N/A	N/A	N/A	N/A	N/A	N/A	N/A	N/A	N/A	N/A
18	**Were the adverse events reported?**	Yes (2) Partial (1) No (0)	N/A	N/A	N/A	N/A	N/A	N/A	N/A	N/A	N/A	N/A	N/A	N/A	N/A	N/A	N/A	N/A	N/A	N/A	N/A	N/A	N/A	N/A
19	**Were the conclusions of the study supported by results?**	Yes (2) Unclear/No (0)	2	2	2	2	2	2	2	2	2	2	2	2	2	2	2	2	2	2	2	2	2	2
**Competing interests and sources of support**																								
20	**Were both competing interests and sources of support for the study reported?**	Yes (2) Partial (1) No (0)	2	1	0	2	0	0	0	1	2	2	0	0	2	0	2	0	0	2	0	0	0	1
	**Total Points (%)**		22/30 (73)	23/32 (72)	16/30 (53)	24/30 (80)	17/30 (57)	21/30 (70)	13/30 (43)	21/30 (70)	19/30 (63)	23/30 (77)	22/30 (73)	17/30 (57)	24/30 (80)	13/30 (43)	22/30 (73)	23/32 (72)	16/30 (53)	23/32 (72)	16/30 (53)	16/30 (53)	20/30 (67)	21/30 (70)
	**Bias Risk (L=Low, M=Medium, H=High)**		M	M	H	L	H	M	H	M	M	M	M	H	L	H	M	M	H	M	H	H	M	M

	**Risk of Bias Assessment for Included Case Reports (1-2 patients)**		**Aziz et al, 2018**	**Black et al, 2014**	**Francis et al, 2007**	**Frebourg et al, 2006**	**Gjyshi et al, 2018**	**Hamilton et al, 2013**	**Herraiz, et al 2012**	**Li et al, 2013**	**Oelschlager et al, 2005**	**Pantelis et al, 2016**	**Shepard et al, 2016**	**Svrcek, 2011**	**van Kouwen et al, 2004**	**Wickremeratne e al, 2014**	**Wilcox et al, 2011**
**1**	**Were patient's demographic characteristics clearly described?**	Yes (2)<br>Partial (1)<br>No (0)	2	2	2	1	2	2	2	2	2	2	2	1	2	2	2
**2**	**Was a patient's history clearly described and presented as a timeline?**	Yes (2)<br>Partial (1)<br>No (0)	1	1	1	1	1	2	2	2	1	1	2	1	2	2	2
**3**	**Was the current clinical condition of the patient on presentation clearly described?**	Yes (2)<br>Partial (1)<br>No (0)	2	2	2	2	2	2	2	2	2	2	2	2	2	2	2
**4**	**Were diagnostic tests or assesment methods and the results clearly described?**	Yes (2)<br>Partial (1)<br>No (0)	2	1	1	1	1	1	1	1	1	1	1	1	2	1	1
**5**	**Was the intervention(s) or treatment procedure(s) clearly described?**	Yes (2)<br>Partial (1)<br>No (0)	2	1	1	1	1	2	2	1	1	1	1	1	1	1	2
**6**	**Was the post-intervention clinical condition clearly described?**	Yes (2)<br>Partial (1)<br>No (0)	0	0	2	1	2	1	1	1	1	1	1	1	2	2	2
**7**	**Were adverse events (harms) or unanticipated events identified and described?**	Yes (2)<br>Partial (1)<br>No (0)	N/A	N/A	N/A	N/A	N/A	N/A	N/A	N/A	N/A	N/A	N/A	N/A	N/A	N/A	N/A
**8**	**Does the case report provide takeaway lessons?**	Yes (2)<br>Unclear/No (0)	2	2	2	2	2	2	2	2	2	2	2	2	2	2	2
	**Total Points (%)**		11/14 (79)	9/14 (64)	11/14 (79)	9/14 (64)	11/14 (79)	12/14 (86)	12/14 (86)	11/14 (79)	10/14 (71)	10/14 (71)	11/14 (79)	9/14 (64)	13/14 (93)	12/14 (86)	13/14 (93)
	**Bias Risk (L=Low, M=Medium, H=High)**		M	M	M	M	M	L	L	M	M	M	M	M	L	L	L

Table S3: Risk of basis assessment for all included case series (Institute of Health Economics for Case Series Studies) and case reports (Joanna Briggs Institute Critical Appraisal Checklist for Case Reports). Citations for included studies are at the end of Table S4.

Reference	Publication Type	Country	Age at Surgery	Sex	*CDH1* Mutation	Endoscopy Protocol Type	Final Endoscopic Biopsy Result	Endoscopic Abnormalties	# Biopsies (Final Scope)	#Biopsies Positive	Postive Biopsy Location	Scopes (n)	Biopsies Total (n)	Scope Adjuncts	Total Surveillance Time (Years)	Last Endo to Surgery (Months)	Total-Embedding Protocol	Blocks Examined (n)	Total foci (n)	Cardia (n)	Fundus (n)	Body (n)	Tansition Zone (n)	Antrum (n)	min diameter foci (mm)	max diameter foci (mm)	*In situ* SRCC	Lymph Nodes Examined (n)	Positive Lymph Nodes (n)	Pagetoid Spread	Staging (TMN)	Follow Up Time (Years)	*H pylori*
Aziz et al, 2018	Case Report	United States	39	F	.	Cambridge	Positive	Normal	30	1	fundus	1	30	.	.	.	.	.	19	0	11	9	0	0	0.5	2.5	0	.	0	.	T1a	.	.
Barber et al, 2008	Case Series	United Kingdom	64	M	45insT	Random	Negative	.	12	0	N/A	.	.	.	8	9	Yes	.	7	1	4	2	0	0	.	.	3	.	0	No	T1a	.	.
			30	F	45insT	Random	Negative	.	24	0	N/A	.	.	.	3	1	Yes	.	24	2	11	11	0	0	.	.	2	.	0	5	T1a	.	.
			36	M	45insT	Random	Positive	.	24	1	antrum	.	.	.	0	2	Yes	.	17	1	5	11	0	0	.	.	1	.	0	1 area	T1a	.	.
			23	F	1466insC	Random	Positive	.	38	1	fundus	.	.	.	0	3	Yes	.	22	0	12	6	4	0	.	.	1	.	0	No	T1a	.	.
			44	F	191C>T	Random	Negative	.	12	0	N/A	.	.	.	0.5	6	Yes	.	0	0	0	0	0	0	.	.	0	.	0	No	No Cancer	.	.
			23	F	641T>C	Random	Positive	.	24	2	fundus,body	.	.	.	0.5	2	Yes	.	16	1	8	7	0	0	.	.	0	.	0	No	T1a	.	.
			20	F	641T>C	Random	Positive	.	24	6	body	.	.	.	0.5	2	Yes	.	66	2	33	16	10	5	.	.	0	.	0	No	T1a	.	.
Bardram et al, 2014	Case Series	Denmark	45	M	602_603delCT	Cambridge	Negative	Normal	30	0	N/A	.	.	.	.	.	Yes	.	.	.	.	.	.	.	.	.	.	12	0	.	T3N0	0.8	.
			41	M	602_603delCT	Cambridge	Positive	Normal	30	1	.	.	.	.	.	.	Yes	.	12	.	.	.	.	.	.	.	.	22	0	.	T1a	0.8	.
			26	F	1565+3insTT	Cambridge	Negative	Normal	30	0	N/A	.	.	.	.	.	Yes	.	5	.	.	.	.	.	.	.	.	16	0	.	T1a	0.5	.
			39	F	1565+3insTT	Cambridge	Positive	Normal	30	2	.	.	.	.	.	.	Yes	.	82	.	.	.	.	.	.	.	.	18	0	.	T1a	0.5	.
			52	F	1565+3insTT	Cambridge	Negative	Normal	30	0	N/A	.	.	.	.	.	Yes	.	16	.	.	.	.	.	.	.	.	16	0	.	T1a	0.4	.
			48	M	1565+3insTT	Cambridge	Positive	Normal	30	1	.	.	.	.	.	.	Yes	.	35	.	.	.	.	.	.	.	.	13	0	.	T1a	0.5	.
			29	M	1565+3insTT	Cambridge	Negative	Normal	30	0	N/A	.	.	.	.	.	Yes	.	72	.	.	.	.	.	.	.	.	41	0	.	T1a	0.4	.
Benesch et al (This publication)	Case Series	Canada	21.7	M	c.2398delC	Random	Positive	gastric polyp	22	2	antrum, body	1	.	.	1	13	Yes	92	45	.	.	.	.	.	.	1	.	28	0	.	T1a	10.0	.
(includes Hebbard et al, 2009 cohort)			22.9	M	c.2398delC	Random	Negative	Normal	16	N/A	N/A	4	.	.	2.2	5	No	57	26	.	.	.	.	.	.	3	2	13	0	.	T1a	8.8	Negative
			23.5	M	c.2398delC	Random	Positive	Erosive gastritis (GE Junction)	20	1	body	2	.	.	1	5	No	49	.	.	.	.	.	.	.	.	.	20	0	.	T1a	10.0	Negative
			25.1	M	1189A>T	Random	Positive	Normal	20	1	body	2	.	.	2.3	6	No	27	22	.	.	.	.	.	.	.	.	14	0	.	T1a	9.0	Negative
			26.8	F	c.2398delC	Random	Positive	Normal	19	1	N/A	2	.	.	1.4	18	Yes	90	52	.	.	.	.	.	.	.	.	21	0	.	T1a	11.9	Positive
			26.9	F	c.2398delC	Random	Positive	Normal	20	1	body	13	.	.	8.8	7	Yes	165	.	.	.	.	.	.	.	.	.	8	0	.	T1a	1.8	Negative
			27.2	F	c.2398delC	Random	Negative	.	11	N/A	N/A	2	.	.	2.1	13	Yes	120	0	.	.	.	.	.	.	.	.	28	0	.	T1a	2.6	Negative
			28.7	F	1189A>T	Random	Negative	Normal	12	N/A	N/A	1	.	.	0.4	12	.	.	.	.	.	.	.	.	.	.	.	.	0	.	T1a	7.7	.
			29.5	F	c.2398delC	Random	Positive	fundic gland polyps	10	1	fundus	10	.	.	5.4	11	No	30	1	.	.	.	.	.	.	.	.	23	0	.	T1a	6.8	Negative
			30.8	F	c.2398delC	Random	Positive	Normal	12	1	body	1	.	.	0.06	2	Yes	75	3	.	.	.	.	.	.	.	.	7	0	.	T1a	13.9	Negative
			31.2	M	c.2398delC	Random	Negative	.	12	N/A	N/A	2	.	.	1.6	20	Yes	195	0	0	0	0	0	0	.	.	.	9	0	.	No Cancer	2.8	Negative
			31.4	M	c.2398delC	Random	Negative	.	.	N/A	N/A	2	.	.	0.3		Yes	114	4	.	.	.	.	.	.	1	.	16	0	.	T1a	5.9	.
			32.1	F	IVS7-2A>G	Random	Negative	.	16	N/A	N/A	1	.	.	5.7	38	Yes	88	7	.	.	.	.	.	.	1	.	9	0	.	T1a	5.9	Negative
			32.9	M	c.2398delC	Random	Negative	Normal	4	N/A	N/A	1	.	.	0.4	5	Yes	164	24	.	.	.	.	.	.	2	.	24	0	.	T1a	9.4	Negative
			35.2	F	c.2398delC	Random	Negative	Normal	12	N/A	N/A	1	.	.	0.8	4	Yes	130	2	.	.	.	.	.	.	.	.	7	0	.	T1a	14.0	Negative
			35.9	M	c.2398delC	Random	Positive	erythema	22	1	antrum	6	.	.	3.6	3	Yes	70	5	.	.	.	.	.	.	2	.	13	0	.	T1a	7.1	Negative
			36.4	M	c.2398delC	Random	Positive	.	12	1	antrum	3	.	.	2.5	5	Yes	106	9	.	.	.	.	.	.	1.4	.	12	0	.	T1a	9.9	Negative
			37.0	M	c.2398delC	Random	Negative	Normal	16	N/A	N/A	1	.	.	0.3	5	Yes	147	3	.	.	.	.	.	.	.	.	4	0	.	T1a	14.4	Negative
			37.1	M	447-453delCAGAAGA	Random	Negative	.	12	N/A	N/A	2	.	.	0.9	44	Yes	82	27	.	.	.	.	.	.	.	.	19	0	.	T1a	11.3	.
			37.7	F	c.2398delC	Random	Negative	hyperemia	30	N/A	N/A	3	.	.	3.1	16	Yes	129	7	.	.	.	.	.	.	2	.	.	0	.	T1a	8.1	Negative
			39.2	M	c.2398delC	Random	Negative	Normal	10	N/A	N/A	5	.	.	9.1	60	Yes	184	5	.	.	.	.	.	.	.	.	9	0	.	T1a	2.1	Negative
			39.8	F	c.2398delC	Random	Positive	flat lesion (body)	16	4	body	1	.	.	1.4	3	No	19	.	.	.	.	.	.	.	20	.	3	0	.	T2	11.9	Negative
			40.6	F	c.2398delC	Random	Positive	Normal	14	1	body	3	.	.	1.9	5	Yes	74	2	.	.	.	.	.	.	.	.	14	0	.	T1a	9.9	Negative
			41.1	F	c.2398delC	Random	Negative	gastritis	2	N/A	N/A	2	.	.	1.5	9	Yes	170	5	.	.	.	.	.	.	.	.	11	0	.	T1a	3.5	Negative
			41.4	F	c.2398delC	Random	Negative	Normal	17	N/A	N/A	1	.	.	0.6	8	Yes	91	7	.	.	.	.	.	.	.	.	7	0	.	T1a	7.7	Negative
			41.7	M	c.2398delC	Random	Negative	Normal	15	N/A	N/A	1	.	.	0.04	1	Yes	110	6	.	.	.	.	.	.	.	.	25	0	.	T1a	13.3	Positive
			43.0	M	c.2398delC	Random	Negative	.	7	N/A	N/A	3	.	.	1.3	1	Yes	107	.	.	.	.	.	.	.	.	.	13	0	.	T1a	13.6	Negative
			43.1	M	c.2398delC	Random	Negative	Normal	12	N/A	N/A	1	.	.	0.5	1	Yes	122	2	.	.	.	.	.	.	.	.	8	0	1	T1a	10.1	.

Reference	Publication Type	Country	Age at Surgery	Sex	*CDH1* Mutation	Endoscopy Protocol Type	Final Endoscopic Biopsy Result	Endoscopic Abnormalties	# Biopsies (Final Scope)	#Biopsies Positive	Postive Biopsy Location	Scopes (n)	Biopsies Total (n)	Scope Adjuncts	Total Surveillance Time (Years)	Last Endo to Surgery (Months)	Total-Embedding Protocol	Blocks Examined (n)	Total foci (n)	Cardia (n)	Fundus (n)	Body (n)	Tansition Zone (n)	Antrum (n)	min diameter foci (mm)	max diameter foci (mm)	*In situ* SRCC	Lymph Nodes Examined (n)	Positive Lymph Nodes (n)	Pagetoid Spread	Staging (TMN)	Follow Up Time (Years)	*H pylori*
			43.2	F	c.2398delC	Random	Negative	Normal	6	N/A	N/A	1	.	.	1.5	15	Yes	103	12	.	.	.	.	.	.	.	.	13	0	.	T1a	13.2	Negative
			45.1	F	c.2398delC	Random	Positive	.	17	1	.	5	.	.	1.2	6	Yes	182	0	0	0	0	0	0	.	.	.	12	0	.	No cancer	3.3	.
			45.8	F	c.2398delC	Random	Negative	Normal	20	N/A	N/A	4	.	.	2.3	9	Yes	247	2	.	.	.	.	.	.	0.4	.	39	0	.	T1s	2.4	Negative
			46.1	F	c.2398delC	Random	Negative	gastritis	20	N/A	N/A	1	.	.	0.1	18	Yes	.	1	.	.	.	.	.	.	.	.	7	0	.	T1a	13.9	Negative
			46.4	F	unknown	Random	Negative	.	3	N/A	N/A	1	.	.	0.1	2	Yes	87	8	.	.	.	.	.	.	.	.	17	0	.	T1a	14.5	Negative
			47.1	F	c.2398delC	Random	Positive	Normal	13	1	antrum	11	.	.	10.3	19	Yes	197	2	.	.	.	.	.	.	1	.	20	0	.	T1a	3.0	Negative
			47.5	F	c.2398delC	Random	Negative	.	14	N/A	N/A	5	.	.	2.4	13	Yes	325	2	.	.	.	.	.	.	9	.	16	0	.	T1a	1.3	Negative
			48.0	F	c.2398delC	Random	Negative	.	16	N/A	N/A	1	.	.	0.7	12	Yes	81	5	.	.	.	.	.	.	.	.	5	0	.	T1a	11.9	Negative
			48.3	F	c.2398delC	Random	Negative	.	15	N/A	N/A	1	.	.	0.5	11	Yes	240	.	.	.	.	.	.	.	3	.	5	0	.	T1a	1.9	Negative
			48.6	M	c.2398delC	Random	Negative	.	1	N/A	N/A	1	.	.	0.2	2	Yes	167	0	0	0	0	0	0	.	.	.	.	0	.	No Cancer	14.2	Negative
			48.9	F	c.2398delC	Random	Negative	Normal	1	N/A	N/A	1	.	.	1.7	10	Yes	184	.	.	.	.	.	.	.	.	.	11	0	.	T1a	2.4	Negative
			49.3	F	c.2398delC	Random	Negative	Normal	15	N/A	N/A	1	.	.	0.2	2	Yes	93	7	.	.	.	.	.	.	.	.	4	0	.	T1a	13.4	Negative
			49.3	M	c.2398delC	Random	Negative	fundal polyp	1	N/A	N/A	1	.	.	0.4	6	Yes	127	1	.	.	.	.	.	.	.	.	7	0	.	T1a	13.8	.
			49.7	F	c.2398delC	Random	Negative	.	16	N/A	N/A	1	.	.	0.4	6	Yes	169	1	.	.	.	.	.	.	.	1	10	0	.	T1a	2.9	Negative
			49.9	F	c.2398delC	Random	Negative	.	.	N/A	N/A	1	.	.	0.5	5	No	50	1	.	.	.	.	.	.	.	.	.	0	.	T1a	3.9	.
			50.0	M	c.2398delC	Random	Negative	.	14	N/A	N/A	1	.	.	1.5	11	Yes	128	18	.	.	.	.	.	.		2	15	0	16	T1a	10.4	Positive
			50.2	F	unknown	Random	Negative	duodenitis	.	N/A	N/A	1	.	.	0.5	7	Yes	120	0	0	0	0	0	0	.	.	.	19	0	.	No Cancer	13.4	Positive
			51.9	F	c.2398delC	Random	Negative	.	13	N/A	N/A	2	.	.	0.6	1	Yes	79	1	.	.	.	.	.	.	.	.	13	0	.	T1a	13.3	Negative
			52.3	M	c.2398delC	Random	Negative	Normal	16	N/A	N/A	1	.	.	0.4	6	Yes	179	3	.	.	.	.	.	.	.	.	23	0	.	T1a	13.3	Negative
			52.8	M	c.2398delC	Random	Negative	gastritis	2	N/A	N/A	1	.	.	2.4	23	Yes	279	.	.	.	.	.	.	.	5	.	8	0	.	T1a	1.7	Negative
			53.6	F	c.2398delC	Random	Negative	.	13	N/A	N/A	3	.	.	1.4	14	Yes	234	0	0	0	0	0	0	.	.	.	6	0	.	No Cancer	3.3	Negative
			55.0	F	unknown	Random	Negative	Normal	7	N/A	N/A	2	.	.	1	5	Yes	.	1	.	.	.	.	.	.	.	.	.	0	.	T1a	4.9	Negative
			55.9	F	c.2398delC	Random	Negative	antral erosions	20	N/A	N/A	1	.	.	5.2	57	No	40	0	0	0	0	0	0	.	.	.	19	0	.	No cancer	6.0	Negative
			56.3	F	c.2398delC	Random	Negative	antrum gastric erosions	17	N/A	N/A	1	.	.	2.4	11	Yes	118	.	.	.	.	.	.	.	.	.	5	0	.	T1a	12.3	Negative
			57.9	M	c.2398delC	Random	Negative	cardia polyp (4 mm)	19	N/A	N/A	1	.	.	0.8	8	Yes	180	0	0	0	0	0	0	.	.	.	14	0	.	No Cancer	2.8	Positive
			58.4	M	c.2398delC	Random	Positive	.	.	1	N/A	2	.	.	1.1	5	Yes	189	1	.	.	.	.	.	.	2	.	9	0	.	T1a	3.8	.
			61.8	F	c.2398delC	Random	Negative	.	.	N/A	N/A	3	.	.	1.3	16	No	14	0	0	0	0	0	0	.	.	.	2	0	.	No Cancer	3.7	.
			63.2	M	c.2398delC	Random	Negative	hyperplastic polups (mid body)	24	N/A	N/A	6	.	.	2.3	4	Yes	92	2	.	.	.	.	.	.	.	.	62	0	.	T1a	11.9	Negative
			63.5	F	c.2398delC	Random	Negative	.	4	N/A	N/A	2	.	.	2.4	9	Yes	161	.	.	.	.	.	.	.	1.5	.	19	0	.	T1a	1.5	Negative
			63.7	M	c.2398delC	Random	Negative	.	7	N/A	N/A	1	.	.	0.3	3	Yes	107	0	0	0	0	0	0	.	.	.	6	0	.	No cancer	8.6	Negative
			72.6	M	c.2398delC	Random	Positive	cardia ulcer (SRCC here)	19	1	cardia	19	.	.	8.3	6	No	21	1	.	.	.	.	.	.	28	.	14	0	.	T1a	5.9	Negative
Black et al, 2014	Case Report	United States	18	.	trp20stop	Random	Negative	Normal	.	0	N/A	3	.	.	.	.	Yes	225	115	.	.	.	.	.	single cells	1.5	6	15	0	.	T1a	.	.
Caron et al, 2008	Case Series	France	22	.	c.2399delG	Random	Negative	Normal	10*	0	N/A	.	.	.	.	.	Yes	100	1	.	.	.	.	.	.	.	1	.	0	.	T1a	.	.
			22	.	c.2399delG	Random	Negative	Normal	10*	0	N/A	.	.	.	.	.	Yes	67	2	.	.	.	.	.	.	.	2	.	0	.	T1a	.	.
			35	.	c.2399delG	Random	Negative	Normal	10*	0	N/A	.	.	.	.	.	Yes	200	2	.	.	.	.	.	.	.	10	.	0	.	T1a	.	.
			30	.	c.2399delG	Random	Negative	Normal	10*	0	N/A	.	.	.	.	.	Yes	88	1	.	.	.	.	.	.	.	1	.	0	.	T1a	.	.
			37	.	c.2399delG	Random	Negative	Normal	10*	0	N/A	.	.	.	.	.	Yes	114	6	.	.	.	.	.	.	.	9	.	0	.	T1a	.	.
			45	.	c.2399delG	Random	Negative	Normal	10*	0	N/A	.	.	.	.	.	Yes	106	4	.	.	.	.	.	.	.	3	.	0	.	T1a	.	.
Castro et al, 2020	Case Series	Portgual	.	.	.	Cambridge	Positive	.	30	3	Antrum/Transition Zone	.	.	.	.	.	Yes	.	.	.	.	.	.	.	.	.	.	.	0		T1b	.	.
			.	.	.	Cambridge	Positive	.	30	1	Cardia	.	.	.	.	.	Yes	.	.	.	.	.	.	.	.	.	.	.	0		T1a	.	.
			.	.	.	Cambridge	Positive	.	30	1	Fundus	.	.	.	.	.	Yes	.	.	.	.	.	.	.	.	.	.	.	0		T1a	.	.
			.	.	.	Cambridge	Negative	.	30	0	N/A	.	.	.	.	.	Yes	.	.	.	.	.	.	.	.	.	.	.	0		T1a	.	.
			.	.	.	Cambridge	Negative	.	30	0	N/A	.	.	.	.	.	Yes	.	.	.	.	.	.	.	.	.	.	.	0		T1a	.	.
			.	.	.	Cambridge	Negative	.	30	0	N/A	.	.	.	.	.	Yes	.	.	.	.	.	.	.	.	.	.	.	0		T1a	.	.
			.	.	.	Cambridge	Negative	.	30	0	N/A	.	.	.	.	.	Yes	.	.	.	.	.	.	.	.	.	.	.	0		T1a	.	.
			.	.	.	Cambridge	Negative	.	30	0	N/A	.	.	.	.	.	Yes	.	.	.	.	.	.	.	.	.	.	.	0		T1a	.	.
			.	.	.	Cambridge	Negative	.	30	0	N/A	.	.	.	.	.	Yes	.	.	.	.	.	.	.	.	.	.	.	0		T1a	.	.
			.	.	.	Cambridge	Negative	.	30	0	N/A	.	.	.	.	.	Yes	.	.	.	.	.	.	.	.	.	.	.	0		T1a	.	.

Reference	Publication Type	Country	Age at Surgery	Sex	*CDH1* Mutation	Endoscopy Protocol Type	Final Endoscopic Biopsy Result	Endoscopic Abnormalties	# Biopsies (Final Scope)	#Biopsies Positive	Postive Biopsy Location	Scopes (n)	Biopsies Total (n)	Scope Adjuncts	Total Surveillance Time (Years)	Last Endo to Surgery (Months)	Total-Embedding Protocol	Blocks Examined (n)	Total foci (n)	Cardia (n)	Fundus (n)	Body (n)	Tansition Zone (n)	Antrum (n)	min diameter foci (mm)	max diameter foci (mm)	*In situ* SRCC	Lymph Nodes Examined (n)	Positive Lymph Nodes (n)	Pagetoid Spread	Staging (TMN)	Follow Up Time (Years)	*H pylori*
			.	.	.	Cambridge	Negative	.	30	0	N/A	.	.	.	.	.	Yes	.	.	.	.	.	.	.	.	.	.	.	0		T1a	.	.
			.	.	.	Cambridge	Negative	.	30	0	N/A	.	.	.	.	.	Yes	.	.	.	.	.	.	.	.	.	.	.	0		T1a	.	.
			.	.	.	Cambridge	Negative	.	30	0	N/A	.	.	.	.	.	Yes	.	.	.	.	.	.	.	.	.	1	.	0		Tis	.	.
Charlton et al, 2004	Case Series	New Zeland	40	M	1008G>T	Random	Positive	1 pale area (body-antrum)	.	.	body-antrum	.	.	Chromo	.	.	No	72	45			22	23	0	0.2	9	.	7	0	.	T1a	.	.
			28	M	1008G>T	Random	Positive	areas (body-antrum)	.	.	body-antrum	.	.	Chromo	.	.	Yes	247	214			123	77	14	0.2	9	.	26	0	.	T1a	.	.
			15	F	1008G>T	Random	Positive	areas (body-antrum)	.	.	body-antrum	.	.	Chromo	.	.	Yes	183	318			257	32	29	0.1	10	.	23	0	.	T1a	.	.
			34	F	1008G>T	Random	Positive	1 pale area (body)	.	.	body	.	.	Chromo	.	.	Yes	207	111			68	23	20	0.1	4	.	15	0	.	T1a		.
			43	F	1792C>T	Random	Negative	Normal	8	.	.	2	.	.	.	14	Yes	271	4			1	3	0	1	3	.	14	0	.	T1a	.	.
			33	F	2287G>T	Random	Positive	ulcer (antrum)	3	.	antrum	2	23	.	.	.	Yes	139	32			16	9	7	0.1	4.5	.	6	0	.	T1a	.	.
Chen et al, 2011	Case Series	United States	53	F	1003C>T	Random	Negative	.	.	.	.	.	.	.	.	.	Yes	118*	17	.	10	3	.	5	.	1.5	3	41	0	.	T1a	4.6	.
details from			52	F	1003C>T	Random	Negative	.	.	.	.	.	.	.	.	.	Yes	118*	16	.	12	3	.	1	.	2.5	0	11	0	.	T1a	4.3	.
Rogers et al,			55	F	1003C>T	Random	Negative	.	.	.	.	.	.	.	.	.	Yes	118*	17	.	17	6	.	1	.	3	7	20	0	.	T1a	4.3	.
2008			50	F	1003C>T	Random	Negative	.	.	.	.	.	.	.	.	.	Yes	118*	15	.	14	3	.	2	.	1.5	4	7	0	.	T1a	4.3	.
details from			56	M	1003C>T	Random	Negative	.	.	.	.	.	.	.	.	.	Yes	118*	4	.	5	0	.	1	.	1	2	16	0	.	T1a	4.1	.
Norton et al,			51	M	1003C>T	Random	Negative	.	.	.	.	.	.	.	.	.	Yes	118*	13	.	10	1	.	3	.	1.5	1	13	0	.	T1a	4.1	.
2008			26	M	1003C>T	Random	Negative	.	.	.	.	.	.	.	.	.	Yes	.	.	.	.	.	.	.	.	.	.	.	0	.	T1a	0.5	.
			70	F	1565+2insT	Random	Positive	.	.	.	.	.	.	.	.	.	Yes	118*	3	.	1	0	.	1	.	4	0	26	0	.	T1a	1.8	.
			18	F	1565+2insT	Random	Negative	.	.	.	.	.	.	.	.	.	Yes	.	.	.	.	.	.	.	.	.	.	.	0	.	T1a	0.1	.
			42	F	c.2398delC	Random	Positive	.	.	.	.	.	.	.	.	.	Yes	118*	2	.	2	0	.	1	.	2.5	1	22	0	.	T1a	3.1	.
			47	M	49-2A>C	Random	Negative	.	.	.	.	.	.	.	.	.	Yes	.	.	.	.	.	.	.	.	.	.	.	0	.	T1a	0.5	.
			50	F	1792C>T	Random	Negative	.	.	.	.	.	.	.	.	.	Yes	.	.	.	.	.	.	.	.	.	.	.	0	.	T1a	0.6	.
			47	F	233C>T	Random	Negative	.	.	.	.	.	.	.	.	.	Yes	.	0	0	0	0	.	0	.	.	.	.	0	.	No Cancer	0.3	.
Chun et al, 2001	Case Series	United States	47	F	1588insC	Random	Negative	.	60	0	N/A	1	60	Chromo	.	.	Yes	.	.	.	.	.	.	.	.	.	.	7	0	.	T1a		Negative
details from			41	F	1588insC	Random	Negative	erythma	.	0	N/A	1	.	.	.	.	Yes	.	1	.	.	.	.	.	.	1	.	2	0	.	T1a		Negative
Carneiro et al,			37	F	1588insC	Random	Negative	.	19	0	N/A	1	19	Chromo	.	.	Yes	.	5	.	.	.	.	.	1	3	.	.	0	.	T1a		Negative
2004			39	M	1588insC	Random	Negative	.	.	0	N/A	1	.	.	3	.	Yes	.	2		.	.	.	.	2	3	.	.	0	.	T1a		Negative
			40	M	1588insC	Random	Negative	.	.	0	N/A	1	.	.	2	.	Yes	.	1	.	.	1	.	.	.	4		.	0	.	T1a		Negative
Devezas et al, 2020	Case Series	Portugal	58	F	c.1901C>T	Cambridge	Negative	Antrum redness	30	0	N/A	.	.	.	.	.	Yes	198	14	.	.	.	.	.	.	.	0	9	0	0	T1a	.	Positive
			42	M	c.1901C>T	Cambridge	Negative	Normal	30	0	N/A	.	.	.	.	.	Yes	370	33	.	.	.	.	.	.	.	2	7	0	2	T1a	.	Positive
details from			43	F	c.1901C>T	Cambridge	Negative	Normal	30	0	N/A	.	.	.	.	.	Yes	261	6	.	.	.	.	.	.	.	0	12	0	0	T1a	.	Positive
Gullo et al, 2008			39	M	c.1901C>T	Cambridge	Negative	Normal	30	0	N/A	.	.	.	.	.	Yes	331	8	.	.	.	.	.	.	.	0	20	0	3	T1a	.	Positive
details from Rocha et al, 2018			30	M	.	Cambridge	Negative	Antrum erosions	30	0	N/A	.	.	.	.	.	Yes	.	2	.	.	.	.	.	.	.	0	.	0	2	T1a	.	.
			18	F	c.1901C>T	Cambridge	Negative	Normal	30	0	N/A	.	.	.	.	.	Yes	170	15	.	.	.	.	.	.	.	0	10	0	0	T1a	.	Positive
			14	F	c.1901C>T	Cambridge	Negative	Antrum pale lesions	30	0	N/A	.	.	.	.	.	Yes	172	3	.	.	.	.	.	0	1.7	0	28	0	1	T1a	.	Negative
			44	F	c.1901C>T	Cambridge	Negative	.	30	0	N/A	.	.	.	.	.	Yes	265	25	.	.	.	.	.	.	.	1	19	0	9	T1a	.	.
			19	M	c.1901C>T	Cambridge	Negative	.	30	0	N/A	.	.	.	.	.	Yes	215	24	.	.	.	.	.	.	.	0	29	0	0	T1a	.	.
			34	M	c.1901C>T	Cambridge	Negative	Normal	30	0	N/A	.	.	.	.	.	Yes	198	14	.	.	.	.	.	.	.	2	.	0	3	T1a	.	.
			51	F	c.1901C>T	Cambridge	Negative	Normal	30	0	N/A	.	.	.	.	.	Yes	253	3	.	.	.	.	.	.	.	0	22	0	0	T1a	.	.
			63	F	c.1901C>T	Cambridge	Negative	Normal	30	0	N/A	.	.	.	.	.	Yes	310	10	.	.	.	.	.	.	.	0	22	0	0	T1a	.	.
			59	M	c.1901C>T	Cambridge	Negative	.	30	0	N/A	.	.	.	.	.	Yes	558	0	0	0	0	0	0	.	.	0	.	0	0	No cancer	.	.
			63	M	c.1901C>T	Cambridge	Negative	Gastric polyp	30	0	N/A	.	.	.	.	.	Yes	.	1	.	.	.	.	.	.	.	0	21	0	0	T1a	.	.
			59	F	.	Cambridge	Negative	Normal	30	0	N/A	.	.	.	.	.	Yes	.	2	.	.	.	.	.	.	.	0	.	0	0	T1a	.	.
			35	F	.	Cambridge	Negative	.	30	0	N/A	.	.	.	.	.	Yes	.	0	.	.	.	.	.	.	.	0	.	0	0	T1a	.	.
			35	F	.	Cambridge	Negative	.	30	0	N/A	.	.	.	.	.	Yes	.	3	.	.	.	.	.	.	.	0	.	0	5	T1a	.	.
			39	F	.	Cambridge	Negative	.	30	0	N/A	.	.	.	.	.	Yes	.	3	.	.	.	.	.	.	.	0	.	0	0	T1a	.	.
			29	M	.	Cambridge	Negative	.	30	0	N/A	.	.	.	.	.	Yes	.	3	.	.	.	.	.	.	.	33		0	0	T1a	.	.
DiBrito et al, 2020	Case Series	United States	24	F	.	Random	Negative	.	.	.	N/A	.	.	.	.	.	.	.	0	0	0	0	0	0	.	.	.	.	.	.	No cancer	.	.
			30	F	.	Random	Negative	.	.	.	N/A	.	.	.	.	.	.	.	0	0	0	0	0	0	.	.	.	.	.	.	No cancer	.	.

Reference	Publication Type	Country	Age at Surgery	Sex	*CDH1* Mutation	Endoscopy Protocol Type	Final Endoscopic Biopsy Result	Endoscopic Abnormalties	# Biopsies (Final Scope)	#Biopsies Positive	Postive Biopsy Location	Scopes (n)	Biopsies Total (n)	Scope Adjuncts	Total Surveillance Time (Years)	Last Endo to Surgery (Months)	Total-Embedding Protocol	Blocks Examined (n)	Total foci (n)	Cardia (n)	Fundus (n)	Body (n)	Tansition Zone (n)	Antrum (n)	min diameter foci (mm)	max diameter foci (mm)	*In situ* SRCC	Lymph Nodes Examined (n)	Positive Lymph Nodes (n)	Pagetoid Spread	Staging (TMN)	Follow Up Time (Years)	*H pylori*
			50	F	.	Random	Negative	.	.	.	N/A	.	.	.	.	.	.	.	0	0	0	0	0	0	.	.	.	.	.	.	No cancer	.	.
			47	F	.	Random	Negative	.	.	.	N/A	.	.	.	.	.	.	.	0	0	0	0	0	0	.	.	.	.	.	.	No cancer	.	.
			25	M	.	Random	Negative	.	.	.	N/A	.	.	.	.	.	.	.	.	.	.	.	.	.	.	.	.	.	.	.	T1a	.	.
			48	F	.	Random	Negative	.	.	.	N/A	.	.	.	.	.	.	.	.	.	.	.	.	.	.	.	.	.	.	.	T1a	.	.
			51	M	.	Random	Negative	.	.	.	N/A	.	.	.	.	.	.	.	.	.	.	.	.	.	.	.	.	.	.	.	Tis	.	.
			49	F	.	Random	Negative	.	.	.	N/A	.	.	.	.	.	.	.	0	0	0	0	0	0	.	.	.	.	.	.	No cancer	.	.
Francis et al, 2007	Case Report	United States	53	F	.	Random	Negative	Normal	.	.	N/A	.	.	.	.	.	Yes	.	11	.	.	.	.	.	.	.	0	.	0	0	T1a	.	.
Frebourg et al, 2006	Case Report	France	20	M	.	Random	Negative	Normal	.	.	N/A	.	.	.	.	.	.	.	80	.	.	.	.	.	.	.	0	.	0	0	T1a	.	.
Fujita et al, 2012	Case Series	United States	38	F	p.R73Q c.2195G>A	Random	Negative	.	.	0	N/A	.	.	.	.	.	Yes	182*	5	.	.	.	.	.	.	3.3	0	13	0	.	T1a	.	.
			41	M	c.1682insA	Random	Negative	.	.	0	N/A	.	.	.	.	.	Yes	182*	7	.	.	.	.	.	.	0.6	5	13	0	.	T1a	.	.
			43	M	c.1901C>T p.A634V	Random	Positive	.	.	.	.	.	.	.	.	.	Yes	182*	136	.	.	.	.	.	.	4	10	16	0	.	T1b	.	.
			42	M	c.48 +1G>A	Random	Negative	.	.	0	N/A	.	.	.	.	.	Yes	182*	11	.	.	.	.	.	.	1.9	4	4	0	.	Tis	.	.
			38	F	c.48 +1G>A	Random	Negative	.	.	0	N/A	.	.	.	.	.	Yes	182*	8	.	.	.	.	.	.	0.1	8	9	0	.	T1a	.	.
			42	M	c.48 +1G>A	Random	Negative	.	.	0	N/A	.	.	.	.	.	Yes	182*	22	.	.	.	.	.	.	2.8	4	16	0	.	T1a	.	.
			50	F	c.1003C>T p.A335X	Random	Negative	.	.	0	N/A	.	.	.	.	.	Yes	182*	8	.	.	.	.	.	.	0.3	1	13	0	.	T1a	.	.
			49	M	c.1003C>T p.A335X	Random	Negative	.	.	0	N/A	.	.	.	.	.	Yes	182*	19	.	.	.	.	.	.	0.3	12	18	0	.	T1a	.	.
			26	M	c.1003C>T p.A335X	Random	Negative	.	.	0	N/A	.	.	.	.	.	Yes	182*	39	.	.	.	.	.	.	1	8	9	0	.	T1a	.	.
			27	F	c.3G>	Random	Negative	.	.	0	N/A	.	.	.	.	.	Yes	182*	47	.	.	.	.	.	.	0.5	37	10	0	.	T1a	.	.
Gjyshi et al, 2018	Case Report	United States	32	M	.	Random	Negative	Normal	.	0	N/A	1	.	.	.	.	Yes	.	3	.	.	.	.	.	.	.	.	30	0	.	T1a	.	.
			23	F	.	Random	Negative	Normal	.	0	N/A	1	.	.	.	.	Yes	.	.	.	.	.	.	.	.	1	.	23	0	.	T1a	.	.
Hackenson et al, 2010	Case Series	United States	50	F	.	Random	Negative	Normal	.	0	N/A	.	.	.	.	.	Yes	.	8.2*	.	.	.	.	.	.	.	.	12.3*	0	.	T1a	.	.
			48	F	.	Random	Negative	Normal	.	0	N/A	.	.	.	.	.	Yes	.	8.2*	.	.	.	.	.	.	.	.	12.3*	0	.	T1a	.	.
			51	M	.	Random	Negative	Normal	.	0	N/A	.	.	.	.	.	Yes	.	8.2*	.	.	.	.	.	.	.	.	12.3*	0	.	T1a	.	.
			21	F	.	Random	Negative	Normal	.	0	N/A	.	.	.	.	.	Yes	.	8.2*	.	.	.	.	.	.	.	.	12.3*	0	.	T1a	.	.
			31	M	.	Random	Negative	Normal	.	0	N/A	.	.	.	.	.	Yes	.	8.2*	.	.	.	.	.	.	.	.	12.3*	0	.	T1a	.	.
			28	M	.	Random	Negative	Normal	.	0	N/A	.	.	.	.	.	Yes	.	8.2*	.	.	.	.	.	.	.	.	12.3*	0	.	T1a	.	.
Hamilton et al, 2013	Case Report	Canada	51	F	.	Random	Negative	Normal	.	0	N/A	2	.	.	.	.	No	50	25	.	.	.	.	.	.	.	.	10	0	.	T1a	.	.
Herraiz et al, 2012	Case Report	Spain	51	F	c.336delC	Random	Negative	Normal	.	0	N/A	1	.	.	.	.	Yes	140	1	.	1	.	.	.	.	0.25	.	.	.		T1a	.	.
Huenburg et al, 2016	Case Series	Germany	23	F	c.1108G>A	Cambridge	Negative	polyp (antrum)	30	0	N/A	1	.	Chromo	0.2	2.3	Yes	.	4	1	0	3	0	0	.	.	.	.	0	.	T1a	0.6	Negative
			61	M	c.1108G>A	Cambridge	Negative	erosion	30	0	N/A	1	.	Chromo	0.2	2.4	Yes	.	5	0	0	5	0	0	.	.	5	.	0	.	Tis	0.5	Negative
			27	M	c.1108G>A	Cambridge	Negative	Normal	30	0	N/A	1	.	Chromo	0.3	3	Yes	.	0	0	0	0	0	0	.	.	.	.	0	.	No Cancer	.	Negative
			39	M	c.1108G>A	Random	Negative	Normal	8	0	N/A	1	.	Chromo	0.4	5	Yes	.	0	0	0	0	0	0	.	.	.	.	0	.	No Cancer	.	Negative
			43	F	c.1108G>A	Cambridge	Negative	polyp (antrum), erythma	30	0	N/A	1	.	Chromo	0.4	5	Yes	.	1	0	0	1	0	0	.	.	.	.	0	.	T1a	2	Negative
			52	F	c.2116C>T	Cambridge	Positive	Normal	30	1	.	1	.	Chromo	0.03	0.4	Yes	.	6	0	4	2	0	0	.	.	.	.	0	.	T1a	0.4	Negative
			54	F	c.2116C>T	Cambridge	Negative	polyp (antrum), erythma	30	0	N/A	1	.	Chromo	0.03	0.4	Yes	.	9	0	4	3	0	2	.	.	.	.	0		T1a	0.7	Negative
			44	F	c.1137G>A	Cambridge	Negative	pale lesion	30	0	N/A	1	.	Chromo	0.003	0.03	Yes	.	2	0	0	2	0	0	.	.	.	.	0	.	T1a	0.5	Negative
Huntsman et al, 2001	Case Series	Canada	35	F	c.1792C>T	Random	Negative	Normal	.	0	N/A	.	.	.	.	15	Yes	140	.	.	.	.	.	.	1	8	.	26	0	.	T1a	.	Negative
details from Carneiro et al, 2004			40	M	c.1711insG	Random	Negative	Normal	.	0	N/A	.	.	.	.	.	Yes	.	.	.	.	.	.	.	.	.	.	.	.	.	T1a	.	Negative
			22	M	c.1711insG	Random	Negative	Normal	.	0	N/A	.	.	.	.	.	Yes	.	.	.	.	.	.	.	.	.	.	.	.	.	T1a	.	Negative
			28	F	c.1711insG	Random	Negative	Normal	.	0	N/A	.	.	.	.	.	Yes	.	.	.	.	.	.	.	.	.	.	.	.	.	T1a	.	Negative
Jacobs et al, 2019	Case Series	United States	28	F	c.1212delC	Cambridge	Negative	Normal	30	0	N/A	.	.	.	.	.	No	.	0	0	0	0	0	0	.	.	.	.	0	.	No cancer	.	.
			24	M	c.1711insG	Cambridge	Negative	Normal	30	0	N/A	.	.	.	.	.	No	.	0	0	0	0	0	0	.	.	.	.	0	.	No cancer	.	.

Reference	Publication Type	Country	Age at Surgery	Sex	*CDH1* Mutation	Endoscopy Protocol Type	Final Endoscopic Biopsy Result	Endoscopic Abnormalties	# Biopsies (Final Scope)	#Biopsies Positive	Postive Biopsy Location	Scopes (n)	Biopsies Total (n)	Scope Adjuncts	Total Surveillance Time (Years)	Last Endo to Surgery (Months)	Total-Embedding Protocol	Blocks Examined (n)	Total foci (n)	Cardia (n)	Fundus (n)	Body (n)	Tansition Zone (n)	Antrum (n)	min diameter foci (mm)	max diameter foci (mm)	*In situ* SRCC	Lymph Nodes Examined (n)	Positive Lymph Nodes (n)	Pagetoid Spread	Staging (TMN)	Follow Up Time (Years)	*H pylori*
			54	F	c.124_126delCCCinsT	Cambridge	Negative	Normal	30	0	N/A	.	.	.	.	.	No	.	.	.	.	.	.	.	.	.	.	.	0	.	T3**	.	.
			28	F	c.124_126delCCCinsT	Cambridge	Positive	Normal	30	.	N/A	.	.	.	.	.	No	.	.	.	.	.	.	.	.	.	.	.	0	.	T1a	.	.
			52	F	c.124_126delCCCinsT	Cambridge	Negative	Normal	30	0	N/A	.	.	.	.	.	No	.	0	0	0	0	0	0	.	.	.	.	0	.	No cancer	.	.
			41	F	c.124_126delCCCinsT	Cambridge	Positive	Normal	30	.	N/A	.	.	.	.	.	No	.	.	.	.	.	.	.	.	.	.	.	0	.	T1a	.	.
			26	F	c.124_126delCCCinsT	Cambridge	Negative	Normal	30	0	N/A	.	.	.	.	.	No	.	.	.	.	.	.	.	.	.	.	.	0	.	T1a	.	.
			45	M	c.1792C>T	Cambridge	Positive	Normal	30	.	N/A	.	.	.	.	.	No	.	.	.	.	.	.	.	.	.	.	.	0	.	T1a	.	.
			46	F	c.1792C>T	Cambridge	Positive	Normal	30	.	N/A	.	.	.	.	.	No	.	.	.	.	.	.	.	.	.	.	.	0	.	T1a	.	.
			63	F	c.1779dupC	Cambridge	Negative	Normal	30	0	N/A	.	.	.	.	.	No	.	0	0	0	0	0	0	.	.	.	.	0	.	No cancer	.	.
			67	M	c.1779dupC	Cambridge	Negative	Normal	30	0	N/A	.	.	.	.	.	No	.	0	0	0	0	0	0	.	.	.	.	0	.	No cancer	.	.
			40	F	c.2430delT	Cambridge	Negative	Normal	30	0	N/A	.	.	.	.	.	No	.	.	.	.	.	.	.	.	.	.	.	0	.	T1a	.	.
			23	F	c.2430delT	Cambridge	Negative	Normal	30	0	N/A	.	.	.	.	.	No	.	.	.	.	.	.	.	.	.	.	.	0	.	T1a	.	.
			46	M	c.2430delT	Cambridge	Positive	Normal	30	.	N/A	.	.	.	.	.	No	.	.	.	.	.	.	.	.	.	.	.	0	.	T1a	.	.
			38	F	c.220C>T	Cambridge	Negative	Normal	30	0	N/A	.	.	.	.	.	No	.	0	0	0	0	0	0	.	.	.	.	0	.	No cancer	.	.
			43	F	c.715G>A	Cambridge	Positive	Normal	30	.	N/A	.	.	.	.	.	No	.	.	.	.	.	.	.	.	.	.	.	0	.	T1a	.	.
Jadot et al, 2019	Case Series	Belgium	41	F	.	Cambridge	Negative	Normal	30	0	N/A	.	.	.	.	.	.	.	.	.	.	.	.	.	.	.	.	.	0	.	Tis	.	.
			68	F	.	Cambridge	Negative	Normal	30	0	N/A	.	.	.	.	.	.	.	0	0	0	0	0	0	.	.	.	.	0	.	No Cancer	.	.
			66	F	.	Cambridge	Negative	Normal	30	0	N/A	.	.	.	.	.	.	.	.	.	.	.	.	.	.	.	.	.	0	.	T1a	.	.
			37	M	.	Cambridge	Negative	Patch	30	0	N/A	.	.	.	.	.	.	.	0	0	0	0	0	0	.	.	.	.	0	.	No Cancer	.	.
			39	M	.	Cambridge	Negative	Patch	30	0	N/A	.	.	.	.	.	.	.	0	0	0	0	0	0	.	.	.	.	0	.	No Cancer	.	.
			41	M	.	Cambridge	Negative	Patch	30	0	N/A	.	.	.	.	.	.	.	0	0	0	0	0	0	.	.	.	.	0	.	No Cancer	.	.
			56	M	.	Cambridge	Negative	Normal	30	0	N/A	.	.	.	.	.	.	.	0	0	0	0	0	0	.	.	.	.	0	.	No Cancer	.	.
			68	F	.	Cambridge	Negative	Patch	30	0	N/A	.	.	.	.	.	.	.	0	0	0	0	0	0	.	.	.	.	0	.	No Cancer	.	.
Khare et al, 2011	Case Series	United States	38*	M	.	Random	Negative	.	.	0	N/A	.	.	.	.	.	.	.	.	.	.	.	.	.	.	.	.	.	0	.	T1a	1.3*	.
			38*	M	.	Random	Negative	.	.	0	N/A	.	.	.	.	.	.	.	.	.	.	.	.	.	.	.	.	.	0	.	T1a	1.3*	.
			38*	M	.	Random	Negative	.	.	0	N/A	.	.	.	.	.	.	.	.	.	.	.	.	.	.	.	.	.	0	.	T1a	1.3*	.
			38*	F	.	Random	Negative	.	.	0	N/A	.	.	.	.	.	.	.	.	.	.	.	.	.	.	.	.	.	0	.	T1a	1.3*	.
			38*	F	.	Random	Negative	.	.	0	N/A	.	.	.	.	.	.	.	.	.	.	.	.	.	.	.	.	.	0	.	T1a	1.3*	.
			38*	F	.	Random	Negative	.	.	0	N/A	.	.	.	.	.	.	.	.	.	.	.	.	.	.	.	.	.	0	.	T1a	1.3*	.
Kumar et al, 2020	Case Series	United States	61	.	c.1565+1G>C	Cambridge	Negative	Polyps (fundus/body)	30	0	N/A	.		EUS	.	.	.	.	8	.	.	.	.	.	1	4	.	.	0	.	T1a	.	.
			28	.	c.1565+1G>C	Cambridge	Negative	Cardia nodule	30	0	N/A	.		EUS	.	.	.	.	.	.	.	.	.	.	.	1	.	.	0	.	T1a	.	.
			40	.	c.1137G>A	Cambridge	Negative	erythema	30	0	N/A	.		EUS	.	.	.	.	1	.	.	.	.	.	.	1	.	.	0	.	T1a	.	.
			40	.	c.199delC	Cambridge	Negative	Normal	30	0	N/A	.		EUS	.	.	.	.	11	.	.	.	.	.	1	15	.	.	0	.	T1a	.	.
			29	.	c.1565+1G>C	Cambridge	Negative	Normal	30	0	N/A	.		EUS	.	.	.	.	67	.	.	.	.	.	1	6	.	.	0	.	T1a	.	.
			25	.	c.603delT	Cambridge	Negative	Normal	30	0	N/A	.		EUS	.	.	.	.	.	.	.	.	.	.	.	1	.	.	0	.	T1a	.	.
			63	.	c.1711+1G>A	Cambridge	Positive	Polyps (cardia)	31	.	Cardia	.		EUS	.	.	.	.	16	.	.	.	.	.	1	4	1	.	0	.	T1a	.	.
			28	.	c.1711+1G>A	Cambridge	Positive	atropic mucosa	30	.	Cardia	.		EUS	.	.	.	.	.	.	.	.	.	.	1	3	.	.	0	.	T1a	.	.
			34	.	c.1711+1G>A	Cambridge	Positive	Cardia nodules	31	.	Cardia	.		EUS	.	.	.	.	.	.	.	.	.	.	1	3	.	.	0	.	T1a	.	.
			35	.	c.1565+1G>C	Cambridge	Positive	Normal	30	.	Cardia	.		EUS	.	.	.	.	.	.	.	.	.	.	.	.	.	.	0	.	T1a	.	.
			54	.	c.603delT	Cambridge	Positive	Normal	30	.	Cardia	.		EUS	.	.	.	.	.	.	.	.	.	.	.	.	.	.	0	.	T2	.	.
			48	.	c.1566-2A>G	Cambridge	Negative	Polyps (fundus)	30	0	N/A	.		EUS	.	.	.	.	0	0	0	0	0	0	.	.	.	.	0	.	No Cancer	.	.
			22	.	c.1566-2A>G	Cambridge	Negative	Normal	30	0	N/A	.		EUS	.	.	.	.	0	0	0	0	0	0	.	.	.	.	0	.	No Cancer	.	.

Reference	Publication Type	Country	Age at Surgery	Sex	*CDH1* Mutation	Endoscopy Protocol Type	Final Endoscopic Biopsy Result	Endoscopic Abnormalties	# Biopsies (Final Scope)	#Biopsies Positive	Postive Biopsy Location	Scopes (n)	Biopsies Total (n)	Scope Adjuncts	Total Surveillance Time (Years)	Last Endo to Surgery (Months)	Total-Embedding Protocol	Blocks Examined (n)	Total foci (n)	Cardia (n)	Fundus (n)	Body (n)	Tansition Zone (n)	Antrum (n)	min diameter foci (mm)	max diameter foci (mm)	*In situ* SRCC	Lymph Nodes Examined (n)	Positive Lymph Nodes (n)	Pagetoid Spread	Staging (TMN)	Follow Up Time (Years)	*H pylori*
Li et al, 2013	Case Report	United States	38	F	c.2287G>T	Random	Negative	Chronic gastritis	.	0	N/A	1	.	.	.	.	.	.	0	0	0	0	0	0	.	.	.	.	0	.	No Cancer	.	.
			32	F	c.2287G>T	Random	Negative	Chronic gastritis	.	0	N/A	1	.	.	.	.	.	.	0	0	0	0	0	0	.	.	.	.	0	.	No Cancer	.	.
Munitiz Ruiz et al, 2019	Case Series	Spain	57	M	c.1577G>A	Random	Negative	Chronic gastritis (antrum)	.	0	.	.	.	.	.	.	.	.	0	0	0	0	0	0	.	.	.	.	0	.	No Cancer	.	.
			32	M	c.1577G>A	Random	Negative	Normal	.	0	.	.	.	.	.	.	.	.	5	.	.	.	.	.	.	.	.	.	0	.	T1a	.	.
			26	F	c.1577G>A	Random	Positive	Normal	.	.	.	.	.	.	.	.	.	.	17	.	.	.	.	.	.	.	.	.	0	.	T1a	.	.
			53	F	c.1577G>A	Random	Negative	Normal	.	0	.	.	.	.	.	.	.	.	6	.	.	.	.	.	.	.	.	.	0	.	T1a	.	.
			43	M	c.1577G>A	Random	Negative	Normal	.	0	.	.	.	.	.	.	.	.	4	.	.	.	.	.	.	.	.	.	0	.	T1a	.	.
			45	F	c.1577G>A	Random	Negative	Normal	.	0	.	.	.	.	.	.	.	.	18	.	.	.	.	.	.	.	.	.	0	.	T1a	.	.
Oelschlarger et al, 2005	Case Report	United States	50	F	.	Random	Negative	Normal	.	0	.	.	.	.	.	.	.	.	.	.	.	.	.	.	.	.	.	13	0	.	T1a	.	.
Pantelis et al, 2016	Case Report	Germany	44	F	c.1137G>A	Random	Negative	Normal	.	0	N/A	.	.	.	.	.	.	.	.	.	.	.	.	.	.	.	.	15	0	.	T1a	.	.
Shepard et al, 2016	Case Report	United States	37	F	.	Random	Negative	Normal	.	0	N/A	.	.	.	.	.	Yes	.	1	.	.	.	.	.	.	0.3	1	6	0	.	T1s	.	.
Svrecek, 2011	Case Report	France	45	F	.	Random	Negative	Normal	.	0	N/A	.	.	.	.	.	.	.	.	.	.	.	.	.	.	.	.	.	0	.	T1a	.	Negative
van Dieren et al, 2020	Case Series	Netherlands	.	.	c.1003C>T	Cambridge	Negative	.	30	0	N/A	1	.	.	.	.	Yes	.	0	0	0	0	0	0	.	.	0	.	0	.	No cancer	.	Negative
			.	.	c.1003C>T	Cambridge	Negative	.	30	0	N/A	1	.	.	.	.	Yes	.	2	0	0	2	0	0	.	.	0	.	0	.	T1a	.	Negative
			.	.	c.1003C>T	Cambridge	Negative	.	30	0	N/A	1	.	.	.	.	Yes	.	0	0	0	0	0	0	.	.	0	.	0	.	No cancer	.	Negative
			.	.	c.1003C>T	Cambridge	Negative	.	30	0	N/A	1	.	.	.	.	Yes	.	0	0	0	0	0	0	.	.	0	.	0	0	No cancer	.	Negative
			.	.	c.1003C>T	Cambridge	Negative	.	30	0	N/A	1	.	.	.	.	Yes	.	1	0	0	0	1	0	.	.	0	.	0	..	T1b	.	Negative
			.	.	c.1003C>T	Cambridge	Positive	.	30	1	Transition Zone	4	.	.	.	.	Yes	.	0	0	0	0	0	0	.	.	0	.	0	.	No cancer	.	Negative
			.	.	c.489C>A	Cambridge	Negative	.	30	0	N/A	1	.	.	.	.	Yes	.	1	0	0	1	0	0	.	.	0	.	0	.	T1a	.	Negative
			.	.	c.489C>A	Cambridge	Negative	.	30	0	N/A	3	.	.	.	.	Yes	.	24	0	0	24	0	0	.	.	0	.	0	.	T1a	.	Negative
			.	.	c.489C>A	Cambridge	Negative	.	30	0	N/A	1	.	.	.	.	Yes	.	4	0	0	4	0	0	.	.	0	.	0	.	T1a	.	Negative
			.	.	c.55_74del	Cambridge	Negative	.	30	0	N/A	1	.	.	.	.	Yes	.	5	0	3	0	0	2	.	.	0	.	0	.	T1a	.	Negative
			.	.	c.55_74del	Cambridge	Negative	.	30	0	N/A	1	.	.	.	.	Yes	.	1	0	0	0	0	0	.	.	0	.	0	.	T1a	.	Negative
			.	.	c.971del	Cambridge	Negative	.	30	0	N/A	1	.	.	.	.	Yes	.	3	0	0	1	0	2	.	.	0	.	0	.	T1a	.	Negative
			.	.	c.971del	Random	Negative	.	.	0	N/A	1	.	.	.	.	Yes	.	14	0	0	14	0	0	.	.	1	.	0	.	T1a	.	Positive
			.	.	c.971del	Cambridge	Negative	.	30	0	N/A	1	.	.	.	.	Yes	.	2	0	0	2	0	0	.	.	0	.	0	.	T1a	.	Negative
			.	.	c.971del	Cambridge	Negative	.	30	0	N/A	1	.	.	.	.	Yes	.	32	5	0	22	1	4	.	.	0	.	0	.	T1a	.	Negative
			.	.	c.971del	Cambridge	Positive	.	30	1	transition zone	1	.	.	.	.	Yes	.	20	0	0	18	0	2	.	.	0	.	0	.	T1a	.	Negative
			.	.	c.971del	Cambridge	Positive	.	30	2	body, fundus	2	.	.	.	.	Yes	.	13	0	0	13	0	0	.	.	0	.	0	.	T1a	.	Negative
			.	.	c.971del	Cambridge	Negative	.	30	0	N/A	4	.	.	.	.	Yes	.	10	0	0	10	0	0	.	.	0	.	0	.	T1a	.	Negative
			.	.	c.971del	Cambridge	Negative	.	30	0	N/A		.	.	.	.	Yes	.	12	0	0	11	1	0	.	.	0	.	0	.	T1a	.	Negative
			.	.	c.971del	Cambridge	Positive	.	30	3	transition Zone, fundus, cardia	1	.	.	.	.	Yes	.	227	1	0	187	20	19	.	.	0	.	0	,	T1a	.	Negative
			.	.	c.2064_2065del	Cambridge	Negative	.	30	0	N/A	1	.	.	.	.	Yes	.	6	0	0	6	0	0	.	.	0	.	0		T1a	.	Positive
			.	.	c.2064_2065del	Cambridge	Negative	.	30	0	N/A	3	.	.	.	.	Yes	.	3	0	0	2	1	0	.	.	0	.	0		T1a	.	Negative
			.	.	c.1565+2dup	Cambridge	Negative	.	30	0	N/A	2	.	.	.	.	Yes	.	12	0	0	11	1	0	.	.	0	.	0		T1a		Negative
			.	.	c.1565+2dup	Cambridge	Positive	.	30	1	fundus	4	.	.	.	.	Yes	.	14	1	0	13	0	0	.	.	0		0		T1a		Negative
			.	.	c.1565+2dup	Cambridge	Negative	.	30	0	N/A	7	.	.	.	.	Yes	.	2	0	0	2	0	0	.	.	0		0		T1a		Negative
			.	.	c.1565+2dup	Cambridge	Negative	.	30	0	N/A	1	.	.	.	.	Yes	.	1	0	0	1	0	0	.	.	0		0		T1a		Negative
			.	.	c.163+2T>A	Cambridge	Positive	.	30	1	Transition zone	1	.	.	.	.	Yes	.	31	0	0	11	5	15	.	.	0		0		T1a		Positive
			.	.	c.163+2T>A	Cambridge	Negative	.	30	0	N/A	1	.	.	.	.	Yes	.	46	1	0	18	0	27	.	.	0		0		T3N0		Positive
			.	.	c.2195G>A	Cambridge	Positive	.	30	2	antrum, fundus	1	.	.	.	.	Yes	.	57	2	0	55	0	0	.	.	0		0		T1a		Positive

Reference	Publication Type	Country	Age at Surgery	Sex	*CDH1* Mutation	Endoscopy Protocol Type	Final Endoscopic Biopsy Result	Endoscopic Abnormalties	# Biopsies (Final Scope)	#Biopsies Positive	Postive Biopsy Location	Scopes (n)	Biopsies Total (n)	Scope Adjuncts	Total Surveillance Time (Years)	Last Endo to Surgery (Months)	Total-Embedding Protocol	Blocks Examined (n)	Total foci (n)	Cardia (n)	Fundus (n)	Body (n)	Tansition Zone (n)	Antrum (n)	min diameter foci (mm)	max diameter foci (mm)	*In situ* SRCC	Lymph Nodes Examined (n)	Positive Lymph Nodes (n)	Pagetoid Spread	Staging (TMN)	Follow Up Time (Years)	*H pylori*
van Kouwen et al, 2004	Case Report	Netherlands	28	F	1135delACG GTTAinsTTA GA	Random	Positive	Normal	.	1	fundus	2	.	.	.	.	.	.	Yes	2	1	.	.	1	.	.	.	19	0	.	T1a	1	.
Wickremeratne et al, 2014	Case Report	Australia	14	F	754dupG	Random	Negative	Normal	.	0	N/A	.	.	.	.	.	Yes	141	19	.	.	.	.	.	0.3	3	.	.	0	.	T1a	.	Negative
Wilcox et al, 2011	Case Report	United States	36	M	1212delC	Random	Negative	Normal	.	0	N/A	.	.	.	.	.	No	40	.	.	.	.	.	.	.	.	.	.	0	.	T1a	.	Negative
**NOT included in Meta-analysis**																																	
Friedman et al, 2019	Case Series	United States	.	M	.	Cambridge		.	30	.		1	30	.	.	.	.	.	.	.	.	.	.	.	.	.	.	.	0	.		.	.
			.	M	.	Cambridge		.	30	.		1	30	.	.	.	.	.	.	.	.	.	.	.	.	.	.	.	0	.		.	.
			.	M	.	Cambridge		.	30	.		1	30	.	.	.	.	.	.	.	.	.	.	.	.	.	.	.	0	.		.	.
			.	M	.	Cambridge		.	30	.		1	30	.	.	.	.	.	.	.	.	.	.	.	.	.	.	.	0	.		.	.
			.	M	.	Cambridge		.	30	.		1	30	.	.	.	.	.	.	.	.	.	.	.	.	.	.	.	0	.		.	.
			.	M	.	Cambridge		.	30	.		1	30	.	.	.	.	.	.	.	.	.	.	.	.	.	.	.	0	.		.	.
			.	F	.	Cambridge		.	30	.		1	30	.	.	.	.	.	.	.	.	.	.	.	.	.	.	.	0	.		.	.
			.	F	.	Cambridge		.	30	.		1	30	.	.	.	.	.	.	.	.	.	.	.	.	.	.	.	0	.		.	.
			.	F	.	Cambridge		.	30	.		1	30	.	.	.	.	.	.	.	.	.	.	.	.	.	.	.	0	.		.	.
			.	F	.	Cambridge		.	30	.		1	30	.	.	.	.	.	.	.	.	.	.	.	.	.	.	.	0	.		.	.
			.	F	.	Cambridge		.	30	.		1	30	.	.	.	.	.	.	.	.	.	.	.	.	.	.	.	0	.		.	.
			.	F	.	Cambridge		.	30	.		1	30	.	.	.	.	.	.	.	.	.	.	.	.	.	.	.	0	.		.	.
			.	F	.	Cambridge		.	30	.		1	30	.	.	.	.	.	.	.	.	.	.	.	.	.	.	.	0	.		.	.
			.	F	.	Cambridge		.	30	.		1	30	.	.	.	.	.	.	.	.	.	.	.	.	.	.	.	0	.		.	.
			.	F	.	Cambridge		.	30	.		1	30	.	.	.	.	.	.	.	.	.	.	.	.	.	.	.	0	.		.	.
			.	F	.	Cambridge		.	30	.		1	30	.	.	.	.	.	.	.	.	.	.	.	.	.	.	.	0	.		.	.
			.	F	.	Cambridge		.	30	.		1	30	.	.	.	.	.	.	.	.	.	.	.	.	.	.	.	0	.		.	.
			.	F	.	Cambridge		.	30	.		1	30	.	.	.	.	.	.	.	.	.	.	.	.	.	.	.	0	.		.	.
			.	F	.	Cambridge		.	30	.		1	30	.	.	.	.	.	.	.	.	.	.	.	.	.	.	.	0	.		.	.
			.	M	.	Cambridge		.	30	.		4.4*	.	.	3.6*	.	.	.	.	.	.	.	.	.	.	.	.	.	0	.		.	.
			.	M	.	Cambridge		.	30	.		4.4*	.	.	3.6*	.	.	.	.	.	.	.	.	.	.	.	.	.	0	.		.	.
			.	M	.	Cambridge		.	30	.		4.4*	.	.	3.6*	.	.	.	.	.	.	.	.	.	.	.	.	.	0	.		.	.
			.	M	.	Cambridge		.	30	.		4.4*	.	.	3.6*	.	.	.	.	.	.	.	.	.	.	.	.	.	0	.		.	.
			.	M	.	Cambridge		.	30	.	fundus	4.4*	.	.	3.6*	.	.	.	.	.	.	.	.	.	.	.	.	.	0	.		.	.
			.	F	.	Cambridge		.	30	.	(2),	4.4*	.	.	3.6*	.	.	.	.	.	.	.	.	.	.	.	.	.	0	.		.	.
			.	F	.	Cambridge		.	30	.	body/fun	4.4*	.	.	3.6*	.	.	.	.	.	.	.	.	.	.	.	.	.	0	.		.	.
			.	F	.	Cambridge		.	30	.	dus (3),	4.4*	.	.	3.6*	.	.	.	.	.	.	.	.	.	.	.	.	.	0	.		.	.
			.	F	.	Cambridge	7	.	30	.	cardia	4.4*	.	.	3.6*	.	.	.	.	.	.	.	.	.	.	.	.	.	0	.	30	.	.
			.	F	.	Cambridge	Positive,	.	30	.	(1),	4.4*	.	.	3.6*	.	.	.	.	.	.	.	.	.	.	.	.	.	0	.	Tis/T1a	.	.
			.	F	.	Cambridge	25	.	30	.	transition	4.4*	.	.	3.6*	.	.	.	.	.	.	.	.	.	.	.	.	.	0	.	, 2 No	.	.
			.	F	.	Cambridge	Negative	.	30	.	zone (1)	4.4*	.	.	3.6*	.	.	.	.	.	.	.	.	.	.	.	.	.	0	.	cancer	.	.
Moslim et al, 2018	Case Series	United States	32*	M	.	Cambridge		.	30	.	.	.	.	.	.	.	.	.	.	.	.	.	.	.	.	.	.	.	0	.		.	.
			32*	M	.	Cambridge		.	30	.	.	.	.	.	.	.	.	.	.	.	.	.	.	.	.	.	.	.	0	.		.	.
			32*	M	.	Cambridge		.	30	.	.	.	.	.	.	.	.	.	.	.	.	.	.	.	.	.	.	.	0	.		.	.
			32*	F	.	Cambridge		.	30	.	.	.	.	.	.	.	.	.	.	.	.	.	.	.	.	.	.	.	0	.		.	.
			32*	F	.	Cambridge		.	30	.	.	.	.	.	.	.	.	.	.	.	.	.	.	.	.	.	.	.	0	.		.	.
			32*	F	.	Cambridge	5	.	30	.	.	.	.	.	.	.	.	.	.	.	.	.	.	.	.	.	.	.	0	.		.	.
			32*	F	.	Cambridge	Positive,	.	30	.	.	.	.	.	.	.	.	.	.	.	.	.	.	.	.	.	.	.	0	.	8 T1a,	.	.
			32*	F	.	Cambridge	4	.	30	.	.	.	.	.	.	.	.	.	.	.	.	.	.	.	.	.	.	.	0	.	1 No	.	.
			32*	F	.	Cambridge	Negative	.	30	.	.	.	.	.	.	.	.	.	.	.	.	.	.	.	.	.	.	.	0	.	Cancer	.	.
Pandalai et al, 2011	Case Series	United States	42	F	R732Q	Random		.	.	.	.	.	.	.	.	.	Yes	.	.	.	.	.	.	.	.	.	.	.	0	.		.	.
			44	M	1682insA	Random		.	.	.	.	.	.	.	.	.	Yes	.	.	.	.	.	.	.	.	.	.	.	0	.		.	.
			46	M	A634V	Random		.	.	.	.	.	.	.	.	.	Yes	.	.	.	.	.	.	.	.	.	.	.	0	.		.	.
			45	M	IVS1+1G>A	Random		.	.	.	.	.	.	.	.	.	Yes	.	.	.	.	.	.	.	.	.	.	.	0	.		.	.
			41	F	IVS1+1G>A	Random		.	.	.	.	.	.	.	.	.	Yes	.	.	.	.	.	.	.	.	.	.	.	0	.		.	.
			43	M	IVS1+1G>A	Random		.	.	.	.	.	.	.	.	.	Yes	.	.	.	.	.	.	.	.	.	.	.	0	.		.	.
			52	F	1003C>TT	Random		.	.	.	.	.	.	.	.	.	Yes	.	.	.	.	.	.	.	.	.	.	.	0	.	1 No	.	.
			50	M	1003C>T	Random	9	.	.	.	.	.	.	.	.	.	Yes	.	.	.	.	.	.	.	.	.	.	.	0	.	Cancer,	.	.
			27	M	1003C>T	Random	Negative,	.	.	.	.	.	.	.	.	.	Yes	.	.	.	.	.	.	.	.	.	.	.	0	.	2 T1a,	.	.
			27	F	3G>A	Random	1 Positive	.	.	.	.	.	.	.	.	.	Yes	.	.	.	.	.	.	.	.	.	.	.	0	.	7 Tis	.	.

Table S4: Data extraction table from included studies. N/A, not applicable. Full references are listed below.

Aziz, M., Madan, R., & Bansal, A. (2018). Hereditary Diffuse Gastric Cancer: More than What Meets the Endoscopic Eye. *Kans J Med, 11*(4), 120-121.
Barber, M. E., Save, V., Carneiro, F., Dwerryhouse, S., Lao-Sirieix, P., Hardwick, R. H., . . . Fitzgerald, R. C. (2008). Histopathological and molecular analysis of gastrectomy specimens from hereditary diffuse gastric cancer patients has implications for endoscopic surveillance of individuals at risk. *J Pathol, 216*(3), 286-294. doi:10.1002/path.2415
Bardram, L., Hansen, T. V. O., Gerdes, A. M., Timshel, S., Friis-Hansen, L., & Federspiel, B. (2014). Prophylactic total gastrectomy in hereditary diffuse gastric cancer: Identification of two novel CDH1 gene mutations - A clinical observational study. *Fam Cancer, 13*(2), 231-242. doi:10.1007/s10689-013-9698-8
Black, M. D., Kaneshiro, R., Lai, J. I., & Shimizu, D. M. (2014). Hereditary diffuse gastric cancer associated with E-cadherin germline mutation: a case report. *Hawai'i journal of medicine & public health : a journal of Asia Pacific Medicine & Public Health, 73*(7), 204-207.
Carneiro, F., Huntsman, D. G., Smyrk, T. C., Owen, D. A., Seruca, R., Pharoah, P., . . . Sobrinho-Simões, M. (2004). Model of the early development of diffuse gastric cancer in E-cadherin mutation carriers and its implications for patient screening. *Journal of Pathology, 203*(2), 681-687. doi:10.1002/path.1564
Caron, O., Schielke, A., Svrcek, M., Fléjou, J.-F., Garzon, J., Olschwang, S., & Sézeur, A. (2008). Usefulness of Prophylactic Gastrectomy in a Novel Large Hereditary Diffuse Gastric Cancer (HDGC) Family. *Official journal of the American College of Gastroenterology | ACG, 103*(8).
Castro, R., Lobo, J., Pita, I., Videira, F., Pedro-Afonso, L., Dinis-Ribeiro, M., & Brandão, C. (2020). Random biopsies in patients harboring a CDH1 mutation: time to change the approach? *Rev Esp Enferm Dig, 112*(5), 367-372. doi:10.17235/reed.2020.6720/2019
Charlton, A., Blair, V., Shaw, D., Parry, S., Guilford, P., & Martin, I. G. (2004). Hereditary diffuse gastric cancer: Predominance of multiple foci of signet ring cell carcinoma in distal stomach and transitional zone. *Gut, 53*(6), 814-820. doi:10.1136/gut.2002.010447
Chen, Y., Kingham, K., Ford, J. M., Rosing, J., Van Dam, J., Jeffrey, R. B., . . . Norton, J. A. (2011). A prospective study of total gastrectomy for CDH1-positive hereditary diffuse gastric cancer. *Ann Surg Oncol, 18*(9), 2594-2598. doi:10.1245/s10434-011-1648-9
Chun, Y. S., Lindor, N. M., Smyrk, T. C., Petersen, B. T., Burgart, L. J., Guilford, P. J., & Donohue, J. H. (2001). Germline E-cadherin gene mutations: Is prophylactic total gastrectomy indicated? *Cancer, 92*(1), 181-187. doi:10.1002/1097-0142(20010701)92:1<181::AID-CNCR1307>3.0.CO;2-J
Devezas, V., Baptista, M., Gullo, I., Rocha, J., Sousa, F., Xiaogang, W., . . . Barbosa, J. (2020). Risk-reducing total gastrectomy in asymptomatic CDH1 carriers: Experience of a tertiary hospital. *European Surgery - Acta Chirurgica Austriaca*. doi:10.1007/s10353-020-00630-7
DiBrito, S. R., Blair, A. B., Prasath, V., Habibi, M., Harmon, J. W., & Duncan, M. D. (2020). Total Gastrectomy for CDH-1 Mutation Carriers: An Institutional Experience. *J Surg Res, 247*, 438-444. doi:10.1016/j.jss.2019.09.062
Francis, W. P., Rodrigues, D. M., Perez, N. E., Lonardo, F., Weaver, D., & Webber, J. D. (2007). Prophylactic laparoscopic-assisted total gastrectomy for hereditary diffuse gastric cancer. *Jsls, 11*(1), 142-147.
Frebourg, T., Oliveira, C., Hochain, P., Karam, R., Manouvrier, S., Graziadio, C., . . . Seruca, R. (2006). Cleft lip/palate and CDH1/E-cadherin mutations in families with hereditary diffuse gastric cancer. *J Med Genet, 43*(2), 138-142. doi:10.1136/jmg.2005.031385
Friedman, M., Adar, T., Patel, D., Lauwers, G. Y., Yoon, S. S., Mullen, J. T., & Chung, D. C. (2019). Surveillance Endoscopy in the Management of Hereditary Diffuse Gastric Cancer Syndrome. *Clinical Gastroenterology and Hepatology*. doi:10.1016/j.cgh.2019.10.033
Fujita, H., Lennerz, J. K., Chung, D. C., Patel, D., Deshpande, V., Yoon, S. S., & Lauwers, G. Y. (2012). Endoscopic surveillance of patients with hereditary diffuse gastric cancer: biopsy recommendations after topographic distribution of cancer foci in a series of 10 CDH1-mutated gastrectomies. *Am J Surg Pathol, 36*(11), 1709-1717. doi:10.1097/PAS.0b013e31826ca204
Gjyshi, O., Vashi, P., Seewald, L., Kohan, M., Abboud, E., Fowler, E., . . . Halabi, H. (2018). Therapeutic and prophylactic gastrectomy in a family with hereditary diffuse gastric cancer secondary to a CDH1 mutation: a case series. *World J Surg Oncol, 16*(1), 143. doi:10.1186/s12957-018-1415-5
Gullo, I., Devezas, V., Baptista, M., Garrido, L., Castedo, S., Morais, R., . . . Carneiro, F. (2018). Phenotypic heterogeneity of hereditary diffuse gastric cancer: report of a family with early-onset disease. *Gastrointest Endosc, 87*(6), 1566-1575. doi:10.1016/j.gie.2018.02.008
Hackenson, D., Edelman, D. A., McGuire, T., Weaver, D. W., & Webber, J. D. (2010). Prophylactic laparoscopic gastrectomy for hereditary diffuse gastric cancer: A case series in a single family. *Journal of the Society of Laparoendoscopic Surgeons, 14*(3), 348-352. doi:10.4293/108680810X12924466007449
Hamilton, L. E., Jones, K., Church, N., & Medlicott, S. (2013). Synchronous appendiceal and intramucosal gastric signet ring cell carcinomas in an individual with CDH1-associated hereditary diffuse gastric carcinoma: a case report of a novel association and review of the literature. *BMC Gastroenterol, 13*, 114. doi:10.1186/1471-230x-13-114
Hebbard, P. C., Macmillan, A., Huntsman, D., Kaurah, P., Carneiro, F., Wen, X., . . . Wirtzfeld, D. A. (2009). Prophylactic total gastrectomy (PTG) for hereditary diffuse gastric cancer (HDGC): the Newfoundland experience with 23 patients. *Ann Surg Oncol, 16*(7), 1890-1895. doi:10.1245/s10434-009-0471-z
Herráiz, M., Valentí, V., Sola, J., Pérez-Rojo, P., Rotellar, F., & Cienfuegos, J. A. (2012). Hereditary diffuse gastric cancer: strategies to reduce tumoral risk. *Rev Esp Enferm Dig, 104*(6), 326-329. doi:10.4321/s1130-01082012000600009
Huneburg, R., Marwitz, T., van Heteren, P., Weismuller, T. J., Trebicka, J., Adam, R., . . . Strassburg, C. P. (2016). Chromoendoscopy in combination with random biopsies does not improve detection of gastric cancer foci in CDH1 mutation positive patients. *Endosc Int Open, 4*(12), E1305-e1310. doi:10.1055/s-0042-112582

Huntsman, D. G., Carneiro, F., Lewis, F. R., MacLeod, P. M., Hayashi, A., Monaghan, K. G., . . . Caldas, C. (2001). Early gastric cancer in young, asymptomatic carriers of germ-line E-cadherin mutations. *N Engl J Med, 344*(25), 1904-1909. doi:10.1056/nejm200106213442504
Jacobs, M. F., Dust, H., Koeppe, E., Wong, S., Mulholland, M., Choi, E. Y., . . . Stoffel, E. M. (2019). Outcomes of Endoscopic Surveillance in Individuals With Genetic Predisposition to Hereditary Diffuse Gastric Cancer. *Gastroenterology, 157*(1), 87-96. doi:10.1053/j.gastro.2019.03.047
Jadot, V., Segers, K., Bours, V., Kohnen, L., Honore, P., Martin, M., . . . Leclercq, P. (2019). [Hereditary diffuse gastric cancer : case serie of 8 patients from a single family and literature review]. *Rev Med Liege, 74*(3), 134-138.
Khare, M., Weaver, D. W., & Hart, J. L. (2011). Case series of prophylactic laparoscopic total gastrectomy for hereditary diffuse gastric cancer with cadherin gene mutation. *Surgical Endoscopy and Other Interventional Techniques, 25*, S302. doi:10.1007/s00464-011-1598-0
Kumar, S., Katona, B. W., Long, J. M., Domchek, S., Rustgi, A. K., Roses, R., & Ginsberg, G. G. (2020). Endoscopic Ultrasound Has Limited Utility in Diagnosis of Gastric Cancer in Carriers of CDH1 Mutations. *Clin Gastroenterol Hepatol, 18*(2), 505-508.e501. doi:10.1016/j.cgh.2019.04.064
Li, J., McBean, E., Li, X., Berho, M., Szomstein, S., & Rosenthal, R. J. (2013). Laparoscopic prophylactic total gastrectomy with linear stapler side-to-side esophagojejunal anastomosis for hereditary diffuse gastric cancer syndrome in 2 siblings. *Surg Laparosc Endosc Percutan Tech, 23*(3), e124-126. doi:10.1097/SLE.0b013e3182773e38
Moslim, M. A., Heald, B., Tu, C., Burke, C. A., & Walsh, R. M. (2018). Early genetic counseling and detection of CDH1 mutation in asymptomatic carriers improves survival in hereditary diffuse gastric cancer. *Surgery, 164*(4), 754-759. doi:10.1016/j.surg.2018.05.059
Munitiz Ruiz, V., Jimeno, P., Ruiz de Angulo, D., Ortiz, A., Martinez de Haro, L. F., Marin, M., . . . Parrilla, P. (2019). Is prophylactic gastrectomy indicated for healthy carriers of CDH1 gene mutations associated with hereditary diffuse gastric cancer? *Rev Esp Enferm Dig, 111*(3), 189-192. doi:10.17235/reed.2018.5831/2018
Norton, J. A., Ham, C. M., Van Dam, J., Jeffrey, R. B., Longacre, T. A., Huntsman, D. G., . . . Ford, J. M. (2007). CDH1 truncating mutations in the E-cadherin gene: an indication for total gastrectomy to treat hereditary diffuse gastric cancer. *Ann Surg, 245*(6), 873-879. doi:10.1097/01.sla.0000254370.29893.e4
Oelschlager, B. K., Yigit, T., Kaufman, J. A., & Pellegrini, C. A. (2005). Hereditary diffuse gastric cancer. *MedGenMed, 7*(3), 16.
Pandalai, P. K., Lauwers, G. Y., Chung, D. C., Patel, D., & Yoon, S. S. (2011). Prophylactic total gastrectomy for individuals with germline CDH1 mutation. *Surgery, 149*(3), 347-355. doi:10.1016/j.surg.2010.07.005
Pantelis, D., Huneburg, R., Adam, R., Holzapfel, S., Gevensleben, H., Nattermann, J., . . . Kalff, J. C. (2016). Prophylactic total gastrectomy in the management of hereditary tumor syndromes. *Int J Colorectal Dis, 31*(12), 1825-1833. doi:10.1007/s00384-016-2656-9
Rocha, J. P., Gullo, I., Wen, X., Devezas, V., Baptista, M., Oliveira, C., & Carneiro, F. (2018). Pathological features of total gastrectomy specimens from asymptomatic hereditary diffuse gastric cancer patients and implications for clinical management. *Histopathology, 73*(6), 878-886. doi:10.1111/his.13715
Rogers, W. M., Dobo, E., Norton, J. A., Van Dam, J., Jeffrey, R. B., Huntsman, D. G., . . . Longacre, T. A. (2008). Risk-reducing total Gastrectomy for germline mutations in E-cadherin (CDH1): Pathologic findings with clinical implications. *American Journal of Surgical Pathology, 32*(6), 799-809. doi:10.1097/PAS.0b013e31815e7f1a
Shepard, B., Yoder, L., & Holmes, C. (2016). Prophylactic Total Gastrectomy for Hereditary Diffuse Gastric Cancer. *ACG case reports journal, 3*(4), e179-e179. doi:10.14309/crj.2016.152
Svrcek, M. (2011). [Case n(o) 6: Signet ring cell intramucosal carcinoma in hereditary diffuse gastric cancer with mutated CDH1 gene]. *Ann Pathol, 31*(5), 381-384. doi:10.1016/j.annpat.2011.07.007
van Dieren, J. M., Kodach, L. L., den Hartog, P., van der Kolk, L. E., Sikorska, K., van Velthuysen, M. F., . . . Cats, A. (2020). Gastroscopic surveillance with targeted biopsies compared with random biopsies in CDH1 mutation carriers. *Endoscopy, 52*(10), 839-846. doi:10.1055/a-1157-8678
Van Kouwen, M. C. A., Drenth, J. P. H., Oyen, W. J. G., De Bruin, J. H. F. M., Ligtenberg, M. J., Bonenkamp, J. J., . . . Nagengast, F. M. (2004). [18F]fluoro-2-deoxy-D-glucose positron emission tomography detects gastric carcinoma in an early stage in an asymptomatic E-cadherin mutation carrier. *Clinical Cancer Research, 10*(19), 6456-6459. doi:10.1158/1078-0432.CCR-04-0599
Wickremeratne, T., Lee, C. H., Kirk, J., Charlton, A., Thomas, G., & Gaskin, K. J. (2014). Prophylactic gastrectomy in a 16-year-old. *Eur J Gastroenterol Hepatol, 26*(3), 353-356. doi:10.1097/meg.0000000000000016
Wilcox, R., Perpich, M., Noffsinger, A., Posner, M. C., & Cooper, K. (2011). Hereditary diffuse gastric cancer: multidisciplinary case report with review of the literature. *Patholog Res Int, 2011*, 845821. doi:10.4061/2011/845821

Variable (SEER Variable)	Variable Name/Description per SEER	Categorization
**Patient ID**		
Patient ID (pubcsnum)	Unique patient ID number, present as a unique entry for each individual cancer diagnosis.	ID for secondary cancers matched to each primary gastric or colorectal cancer diagnosis.
**Exposure**		
Cancer Type (siterwho)	Gastric siterwho = (21020)<br>Colorectal siterwho = [(21041 (Cecum), 21043, (Ascending Colon), 21044 (Hepatic Flexure), 21045 (Transverse Colon), 21046 (Splenic Flexure), 21047 (Descending Colon), 21048 (Sigmoid Colon), 21049 (Large Intestine, NOS), 21051 (Rectosigmoid junction), 21052 (Rectum)]<br>Breast siterwho =(26000)	Gastric – 21020<br>Colorectal – (21041, 21043, 21044, 21045, 21046, 21047, 21048, 21049, 21051, 21052)<br>Breast – 26000
Histology (ICD-0-3) (histo3v)	849x/x (Signet Ring Cell Adenocarcinoma)<br>814x/x (Adenocarcinoma, NOS)<br>850x/x (Ductal Carcinoma) (Breast)<br>852x/x (Lobular and other Ductal Cancer) (Breast)	Signet Ring – 849x<br>Adenocarcinoma – 814x<br>Ductal – 850x<br>Lobular – 852x
**Outcome**		
Follow-up Time (srv_time_mon)	srv_time_mon (Survival months)<br>0-9998 (in months), 9999 (Unknown)	Months (0-9998)
**Competing Risk**		
Mortality (from primary cancer) (vsrtsadx)	vsrtsadx (SEER cause-specific death classification)<br>0 (Alive or dead of other cause), 1 (Dead), 8 (Dead – missing/unknown cause of death), 9 (N/A not first tumor)	Alive/N/A – 0,9<br>Dead – 1
**Co-variables**		
Age (age_dx)	000-130 (Actual age of diagnosis in years), 999 (Unknown)	Age (0-130 years)
Gender (sex)	1 (Male), 2 (Female)	Male – 1<br>Female – 0

Table S5. Variable definition in SEER secondary cancer analysis.

Primary Cancer	Gastric		Colorectal	
**Data Processing Scheme**	**Count (All Primary Cancer Cases)**	**Count (All Secondary Cancer Cases)**	**Count (All Primary Cancer Cases)**	**Count (All Secondary Cancer Cases)**
Initial count of all cases.	174,763	32,240	1,078,276	228,398
Drop if age_dx is unknown (age_dx=999).	174,749	32,340	1,078,221	228,397
Drop if sex is unknown.	174,749	32,340	1,078,221	228,397
Drop if survival months is unknown (srv_time_mon=9999).	172,375	32,056	1,068,086	227,305

Table S6. Exclusion criteria and counts of all cases in SEER secondary cancer analysis.

**Cancer Type (N)**	**Age**
Lobular breast cancer (9)<br>1 case with SRCC	53 (38-60) (mean, range)<br>54
Colon (SRCC)	46
Rectal	51
Melanoma	68
Pancreatic	65
B-cell lymphoma (Non-Hodgkin)	62
Multiple Myeloma	81
Basal Cell	73
Prostate	unknown

Table S7: Secondary cancers observed in Newfoundland and Labrador *CDH1* mutation cohort. SRCC (signet ring cell adenocarcinoma).
